# Supplementary material for: Families with neurodevelopmental diagnoses are not ‘Hard to Reach’: Findings from a feasibility trial comparing parenting programmes for parents of children with ADHD
Source: PLoS One. 2025 Sep 17;20(9):e0323959. doi: 10.1371/journal.pone.0323959 (PMC12443243; doi:10.1371/journal.pone.0323959)
Supplement: S1 Table — (DOCX) [file pone.0323959.s001.docx]

| Table S1. Between-group difference secondary outcome measures | | | | |
| --- | --- | --- | --- | --- |
|  |  | IY vs. PInC | | |
| Measure | Subscale | Adjusted mean difference | (95% CI) | p-value |
|  |  |  |  |  |
| ECBI | Prob | 2.12 | -5.48, 9.71 | 0.594 |
|  | Int | 7.77 | -16.04, 31.59 | 0.533 |
| SDQ | Emo | 0.89 | -1.75, 3.53 | 0.519 |
|  | Con | 0.94 | -0.71, 2.60 | 0.284 |
|  | Hyp | -0.49 | -1.85, 0.87 | 0.494 |
|  | PP | 1.28 | -1.38, 3.95 | 0.362 |
|  | TDS | 2.80 | -2.71, 8.32 | 0.338 |
| GHQ |  | -2.56 | -9.99, 4.88 | 0.512 |
| ASRS | Part A | -1.95 | -5.55, 1.65 | 0.305 |
|  | Part B | 2.55 | -4.99, 10.08 | 0.518 |
| PDHS | CB-I | 4.58 | -1.04, 10.21 | 0.134 |
|  | CB-F | 2.98 | 0.05, 5.92 | 0.068 |
|  | PT-I | 2.36 | -1.51, 6.23 | 0.253 |
|  | PT-F | 0.61 | -2.37, 3.60 | 0.693 |
| EQ-5D |  | 0.03 | -0.16, 0.23 | 0.744 |
| PEDS-QL | Psy-Soc | -6.18 | -17.00, 4.63 | 0.281 |
|  | Phys | 2.85 | -13.77, 19.48 | 0.742 |
|  | Total | -2.62 | -13.53, 8.29 | 0.645 |

Key: IY: Incredible Years; PInC: Parents InC; CI: Confidence interval; ECBI: Eyberg Child Behaviour Inventory; Prob: Problems subscale; Int: Intensity subscale; SDQ: Strengths and Difficulties Questionnaire; Emo: Emotional Problems subscale; Con: Conduct Problems subscale; Hyp: Hyperactivity-inattention subscale; PP: Peer Problems subscale; TDS: Total Difficulties Score; GHQ: General Health Questionnaire; ASRS: ADHD Symptom Rating Scale-version 1; PDHS: Parenting Daily Hassles Scale; CB-I: Child Behaviour – Intensity; CB-F: Child Behaviour – Frequency; PT-I: Parenting Tasks – Intensity; PT-F: Parenting Tasks – Frequency; EQ-5D: EuroQol-5 Dimensions, five level version; PEDS-QL: Pediatric Quality of Life Inventory; Psy-Soc: Psychosocial Health Summary Score; Phys: Physical Health Summary Score.
