# Supplementary material for: Families with neurodevelopmental diagnoses are not ‘Hard to Reach’: Findings from a feasibility trial comparing parenting programmes for parents of children with ADHD
Source: PLoS One. 2025 Sep 17;20(9):e0323959. doi: 10.1371/journal.pone.0323959 (PMC12443243; doi:10.1371/journal.pone.0323959)
Supplement: S2 File — (DOCX) [file pone.0323959.s002.docx]

**Support for PARents of Children Living with ADHD – a Research trial**

**SPARCLAR**

**STATISTICAL ANALYSIS PLAN (SAP)**

| Study Title | **S**upport for **PAR**ents of **C**hildren **L**iving with **A**DHD – a **R**esearch trial | | | | |
| --- | --- | --- | --- | --- | --- |
| Short Title | SPARCLAR | | | | |
| IDs | Sponsor reference 300396  CSO reference HIPS/17/58  REC number 18/NS/0124  IRAS number 248214 | | | | |
| Sponsor | University of Glasgow | | | | |
| Funded by | CSO |  |  |  |  |
| Protocol Version | 5 | Date | 07/05/2020 |  |  |
| SAP Version | 1.0 | Date | 28/10/2020 |  |  |
|  |  |  |  |  |  |
|  |  |  | Signature |  | Date |
|  |  |  |  |  |  |
| Prepared by | Dr Martina Messow | | 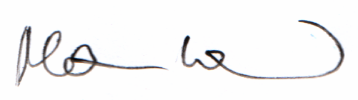 |  | 28/10/2020 |
|  | Robertson Centre for Biostatistics  University of Glasgow | | | | |
|  |  |  | 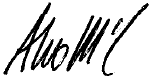 |  |  |
|  |  |  |  |  |  |
| Approved by | Prof Alex McConnachie | |  |  | 28/10/2020 |
|  | Robertson Centre for Biostatistics  University of Glasgow | | | | |
|  |  |  |  |  |  |
|  |  |  |  |  |  |
|  | Prof Helen Minnis | | 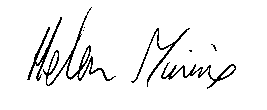 |  | 05/11/2020 |
|  | Academic CAMHS, Institute of Health and Wellbeing, Level 4, West Glasgow Ambulatory Care Hospital, Yorkhill, Glasgow, G3 8SJ | | | | |
|  |  |  |  |  |  |
|  |  |  |  |  |  |
|  | Dr Lucy Thompson | | 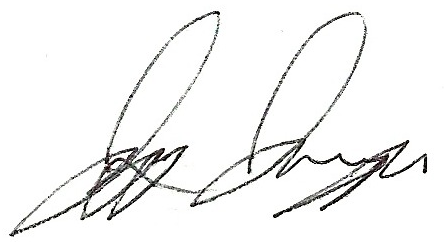 |  | 05/11/2020 |
|  | Academic CAMHS, Institute of Health and Wellbeing, Level 4, West Glasgow Ambulatory Care Hospital, Yorkhill, Glasgow, G3 8SJ | | | | |
|  |  |  |  |  |  |

CONTENTS

[1 Introduction 3](#_Toc54717356)

[1.1 Study Background 3](#_Toc54717357)

[1.2 Study Objectives 3](#_Toc54717358)

[1.3 Study Design 3](#_Toc54717359)

[1.4 Randomisation 3](#_Toc54717360)

[1.5 Sample Size and Power 3](#_Toc54717361)

[1.6 Interim Analyses and Stopping Rules 3](#_Toc54717362)

[1.7 Study Population 4](#_Toc54717363)

[1.7.1 Inclusion criteria 4](#_Toc54717364)

[1.7.2 Exclusion criteria 4](#_Toc54717365)

[1.8 Feasibility Outcomes 4](#_Toc54717366)

[1.9 Proposed Efficacy Outcomes 4](#_Toc54717367)

[1.9.1 Primary Outcome 4](#_Toc54717368)

[1.9.2 Secondary Outcomes 4](#_Toc54717369)

[1.10 Statistical Analysis Plan (SAP) 5](#_Toc54717370)

[1.10.1 SAP Objectives 5](#_Toc54717371)

[1.10.2 General Principles 5](#_Toc54717372)

[1.10.3 Current Protocol 5](#_Toc54717373)

[1.10.4 Deviations from Those Specified in Study Protocol 5](#_Toc54717374)

[1.10.5 Additional Analyses to Those Specified in Study Protocol 5](#_Toc54717375)

[1.10.6 Software 6](#_Toc54717376)

[2 Analysis populations 6](#_Toc54717377)

[2.1 Efficacy 6](#_Toc54717378)

[2.2 Safety 6](#_Toc54717379)

[3 Analysis 6](#_Toc54717380)

[3.1 Subject disposition 6](#_Toc54717381)

[3.2 Baseline characteristics 6](#_Toc54717382)

[3.3 Feasibility outcomes 7](#_Toc54717383)

[3.4 Efficacy Outcomes 7](#_Toc54717384)

[3.4.1 Putative Primary Outcome 7](#_Toc54717385)

[3.4.2 Putative Secondary outcomes 7](#_Toc54717386)

[3.4.3 Additional Analyses 7](#_Toc54717387)

[3.4.4 Subgroup Analyses 7](#_Toc54717388)

[3.4.5 Sensitivity Analyses 7](#_Toc54717389)

[3.4.6 Per protocol analysis 8](#_Toc54717390)

[3.5 Safety Outcomes 8](#_Toc54717391)

[3.5.1 Treatment Compliance 8](#_Toc54717392)

[3.5.2 Premature Withdrawal 8](#_Toc54717393)

[3.5.3 Adverse Events 8](#_Toc54717394)

[3.5.4 Concomitant Medications 8](#_Toc54717395)

[4 Data Conventions 8](#_Toc54717396)

[5 Document History 8](#_Toc54717397)

[6 References 8](#_Toc54717398)

[7 Table Templates 9](#_Toc54717399)

# Introduction

## Study Background

Attention Deficit/Hyperactivity Disorder (ADHD) substantially burdens affected children, parents and society. It is well-known that early drug therapies can change trajectories and reduce individual, family and societal burden, and cost‐effectiveness studies have focused on available drug therapies and less on non‐medical interventions. Children with neurodevelopmental problems such as ADHD are at higher risk of experiencing abuse and/or neglect so it is crucial that their families receive targeted support. As a first line of treatment, NICE guidelines recommend a group parenting intervention, the effectiveness of which has been proven. It is however unclear whether an intervention designed specifically for families of children with a diagnosis will be more effective and cost effective.

Parents In Control (Parents InC / PInC) offers support designed specifically for parents of children with ADHD. The current best evidenced alternative is Incredible Years (IY). What needs to be determined is whether PInC i) is at least as effective as IY in impacting children’s behaviour outcomes; ii) is cost‐effective; and iii) offers something unique in terms of parenting self‐competence and quality of life.

## Study Objectives

This study is a feasibility trial aiming to address the following research questions:

1. Are parents of children recently diagnosed with ADHD willing to be randomised to PInC or IY?
2. Can sufficient numbers of families be (a) recruited and (b) retained such that a full‐scale RCT is likely to be feasible?
3. Are research procedures and measures feasible and acceptable to participating families?

## Study Design

A randomised controlled feasibility trial. The intervention will be delivered in four waves. Participants of waves 1, 2 and 3 will have follow up assessments after 12 months, participants of wave 4 will have follow-up assessments after 6 months.

## Randomisation

Families will be randomised 1:1 to receive either PInC or IY. No stratification or minimisation is applied.

## Sample Size and Power

In an unfunded previous evaluation there were 462 children referred to PInC in 4 years in Fife, with 228 (49%) attending at least the first session. In funded intervention studies in similar populations, response and retention rates have been higher. On this basis, the aim will be to approach 100 eligible participants over 7-12 months. Assuming a 60% response rate and two-thirds retention, this will result in 20 families in each intervention arm.

## Interim Analyses and Stopping Rules

There are no planned interim analyses or stopping rules in this trial.

## Study Population

### Inclusion criteria

Parents of children aged 5-12 with a formal diagnosis of ADHD, following a standardised assessment from a paediatrician or a psychiatrist through the Fife integrated ADHD pathway during the one-year recruitment period.

### Exclusion criteria

1. Family attending other parenting groups.
2. Inability to complete questionnaires or participate in groups because of limited English language comprehension.
3. Participants already taking part in research on a parenting intervention.

## Feasibility Outcomes

The outcomes related to feasibility are

- Consent rate among all families that were approached;
- Retention rate among all consenting families;
- Completeness of the putative outcome measures for a full trial.

## Proposed Efficacy Outcomes

### Primary Outcome

The proposed primary outcome for a full trial is the parental sense of competence, measured by the Parental Sense of Competence Scale (PSOC).

### Secondary Outcomes

The proposed secondary outcomes for a full trial are

- Eyberg Child Behaviour Inventory (ECBI) problem score > 11 / intensity score > 127
- Strength and Difficulties Questionnaire (SDQ) total score
- General Health Questionnaire (GHQ)
- Parental ADHD symptoms measured using the ADHD Rating Scale (ASRS-v1.1) Part A and Part B
- Parenting stress measured using the Parenting Daily Hassles Scale (PDHS) challenging behaviour intensity and frequency , parenting tasks frequency and intensity
- Goal Based Outcomes from the Child Outcomes Research Consortium (CORC-GBO) – amount of movement along the scale from baseline to follow-up
- Parent quality of life measured using the EQ-5D-5L (health utility score and VAS)
- Child quality of life measured using the PedsQL total score, psychosocial health summary score, physical health summary score
- Service use

## Statistical Analysis Plan (SAP)

### SAP Objectives

The objective of this SAP is to describe the statistical analyses to be carried out for the SPARCLAR study.

### General Principles

Rates will be calculated with Clopper-Pearson^1^ 95% confidence intervals.

Proposed primary and secondary outcome measures will be summarised at baseline and follow up, and separately by follow-up time point (12 months for recruitment waves 1 to 3, 6 months for recruitment wave 4). Changes from baseline in the proposed outcome measures will be summarised in the same way. All summaries will include the number of observed values and the number of missing values. Continuous variables will be summarised by the mean, standard deviation, median, interquartile range, minimum and maximum. Categorical variables will be summarised as the number and percentage of participants in each group.

The primary and numeric secondary efficacy outcomes will be compared between intervention groups using linear regression analyses predicting change in outcome from intervention group, adjusting for baseline value of the outcome, age and gender.

Binary secondary efficacy outcomes will be compared between intervention groups using logistic regression analyses predicting outcome from intervention group, adjusting for age and gender.

If numbers are too low for the planned regression analysis, unadjusted regression analyses or simple tests will be carried out instead. For numbers below five in any group, no statistical tests will be carried out.

The distribution of the residuals of each linear regression analysis will be reviewed and transformations will be applied to the outcome variable as appropriate.

Regression results will be reported as adjusted mean difference between intervention groups, or adjusted odds ratios for logistic regressions, with 95% confidence interval and p-value.

Missing values will not be imputed.

Health economic analyses will be carried out by health economists and are not part of this SAP.

### Current Protocol

The current study protocol at the time of writing is version 5, dated 07/05/2020. Future amendments to the protocol will be reviewed for their impact on this SAP, which will be updated only if necessary. If no changes are required to this SAP following future amendments to the study protocol, this will be documented as part of the Robertson Centre Impact Assessment process.

### Deviations from Those Specified in Study Protocol

No planned deviations from analyses planned in the study protocol.

### Additional Analyses to Those Specified in Study Protocol

No planned additional analyses to those planned in the study protocol.

### Software

Data will be analysed using R version 3.6.2 or SAS version 9.4 or higher versions of those programs.

# Analysis populations

## Efficacy

The intention-to-treat (ITT) population will consist of all participants who consented and provided any baseline data.

The per-protocol (PP) population will consist of all participants who consented and provided any baseline data and attended at least seven out of 14 sessions for IY or two out of six sessions for PInC.

## Safety

The safety population will consist of all participants who consented.

# Analysis

## Subject disposition

The number of participants in the ITT population and the number of participants in the safety population will be tabulated.

## Baseline characteristics

The following baseline characteristics relating to the child will be summarised in the ITT population:

- Age
- Gender
- Time since diagnosis
- Relationship with main carer
- Being on ADHD medication
- Time since starting ADHD medication (earliest recorded if more than one)
- Other neurodevelopmental diagnosis

The following baseline characteristics relating to the parent will be summarised in the ITT population:

- Age
- Gender
- SIMD
- Relationship with child
- Ethnicity
- Education
- Average household income

## Feasibility outcomes

The following proportions will be calculated:

- the proportion of eligible subjects that was approached;
- the proportion of approached subjects who consented to take part in the trial;
- the proportion of approached subjects who consented to take part in the trial and provide any baseline data;
- the proportion of consented participants who provide any follow-up data;
- the proportion of consented participants in wave 1, 2 and 3 who provide any follow-up data at 12 months;
- the proportion of consented participants in wave 4 who provide any follow-up data at 6 months;
- the proportion of consented participants who provide outcome data for each of the efficacy outcomes separately, overall and by waves 1-3 vs. wave 4.

## Efficacy Outcomes

### Putative Primary Outcome

Change from baseline in PSOC will be analysed in a linear regression analysis predicting change from baseline from randomised intervention group adjusting for baseline PSOC, child age and gender.

### Putative Secondary outcomes

Change from baseline in each continuous secondary outcome measure will be analysed in a linear regression analysis predicting change from baseline from randomised intervention group adjusting for baseline value of the outcome measure, child age and gender.

Binary secondary outcome measures (ECBI problem score > 11 and ECBI intensity score > 127) will be analysed in a logistic regression analysis predicting the outcome from randomised intervention group adjusting for baseline ECBI problem score or ECBI intensity score (as continuous measures), child age and gender.

### Additional Analyses

No additional analyses planned.

### Subgroup Analyses

To assess the impact of the follow-up time on the proposed primary and secondary outcomes, the analysis will be repeated in those participants who have 12-month follow-up only.

### Sensitivity Analyses

The following measures will be taken at baseline and repeated prior to starting the intervention if the waiting time exceeds 3 months, to assess to what extent baseline measures are changing over time and how this affects the results:

- SDQ
- PDHS
- GBO
- EQ-5D-5L

As a sensitivity analysis, the secondary analyses relating to those outcomes will be repeated using the repeat baseline measure instead of the baseline measure.

### Per protocol analysis

The analysis of the primary outcome will be repeated using treatment received instead of allocated treatment in the PP population.

## Safety Outcomes

### Treatment Compliance

Group attendance will be summarised overall and by intervention group.

### Premature Withdrawal

Withdrawals and withdrawal reasons will be summarised overall and by intervention group.

### Adverse Events

Information is collected on Serious Adverse Events only. The number of participants experiencing at least one SAE will be summarised overall and by intervention group. SAE characteristics (seriousness criteria, severity, expectedness, relation to intervention, outcome, duration) will be summarised overall and by study group. All SAEs will be listed.

### Concomitant Medications

Parent and child medication use will be summarised overall and by intervention group at baseline and follow-up for medications of interest. These will be specified prior to the final analysis.

# Data Conventions

A separate assumptions document named SPARCLAR_Assumptions_vx_x.docx detailing data rules (e.g. partial dates) will be agreed prior to database lock.

# Document History

This is version 1.0 of the SAP for the SPARCLAR study, its initial creation, dated 28/10/2020.

# References

[1] Clopper, C. J. & Pearson, E. S. (1934). The use of confidence or fiducial limits illustrated in the case of the binomial. Biometrika, **26**, 404–413. doi: [10.2307/2331986](https://doi.org/10.2307/2331986).

# Table Templates

Table 1.1 Recruitment. Proportions reported in % with 95% confidence interval.

|  | N | Proportion |
| --- | --- | --- |
| Eligible subjects in research period | nn |  |
| Approached subjects (out of all eligible subjects) | nn | xx.x (xx.x – xx.x) |
| Subjects consented (out of all subjects approached) | nn | xx.x (xx.x – xx.x) |
| Subjects consented with baseline data (out of all subjects approached) | nn | xx.x (xx.x – xx.x) |

Table 1.2 Retention. Proportions are out of all who consented and have any baseline data (ITT population), reported in % with 95% confidence interval.

|  |  | | Randomised intervention | | | |
| --- | --- | --- | --- | --- | --- | --- |
|  | All | | PInC | | IY | |
|  | N | Proportion | N | Proportion | N | Proportion |
| Randomised | nn |  | nn |  | Nn |  |
| Provide any follow-up data | nn | xx.x (xx.x – xx.x) | nn | xx.x (xx.x – xx.x) | nn | xx.x (xx.x – xx.x) |
| Provide follow-up data for |  |  |  |  |  |  |
| PSOC | nn | xx.x (xx.x – xx.x) | nn | xx.x (xx.x – xx.x) | nn | xx.x (xx.x – xx.x) |
| ECBI problem score | nn | xx.x (xx.x – xx.x) | nn | xx.x (xx.x – xx.x) | nn | xx.x (xx.x – xx.x) |
| ECBI intensity score | nn | xx.x (xx.x – xx.x) | nn | xx.x (xx.x – xx.x) | nn | xx.x (xx.x – xx.x) |
| SDQ | nn | xx.x (xx.x – xx.x) | nn | xx.x (xx.x – xx.x) | nn | xx.x (xx.x – xx.x) |
| GHQ | nn | xx.x (xx.x – xx.x) | nn | xx.x (xx.x – xx.x) | nn | xx.x (xx.x – xx.x) |
| ASRS (parent) Part A | nn | xx.x (xx.x – xx.x) | nn | xx.x (xx.x – xx.x) | nn | xx.x (xx.x – xx.x) |
| ASRS (parent) Part B | nn | xx.x (xx.x – xx.x) | nn | xx.x (xx.x – xx.x) | nn | xx.x (xx.x – xx.x) |
| PDHS challenging behaviour intensity | nn | xx.x (xx.x – xx.x) | nn | xx.x (xx.x – xx.x) | nn | xx.x (xx.x – xx.x) |
| PDHS challenging behaviour frequency | nn | xx.x (xx.x – xx.x) | nn | xx.x (xx.x – xx.x) | nn | xx.x (xx.x – xx.x) |
| PDHS parenting tasks intensity | nn | xx.x (xx.x – xx.x) | nn | xx.x (xx.x – xx.x) | nn | xx.x (xx.x – xx.x) |
| PDHS parenting tasks frequency | nn | xx.x (xx.x – xx.x) | nn | xx.x (xx.x – xx.x) | nn | xx.x (xx.x – xx.x) |
| CORC-GBO | nn | xx.x (xx.x – xx.x) | nn | xx.x (xx.x – xx.x) | nn | xx.x (xx.x – xx.x) |
| EQ-5D-5L | nn | xx.x (xx.x – xx.x) | nn | xx.x (xx.x – xx.x) | nn | xx.x (xx.x – xx.x) |
| PedsQL psychosocial health summary score | nn | xx.x (xx.x – xx.x) | nn | xx.x (xx.x – xx.x) | nn | xx.x (xx.x – xx.x) |
| PedsQL physical health summary score | nn | xx.x (xx.x – xx.x) | nn | xx.x (xx.x – xx.x) | nn | xx.x (xx.x – xx.x) |
| PedsQL total score | nn | xx.x (xx.x – xx.x) | nn | xx.x (xx.x – xx.x) | nn | xx.x (xx.x – xx.x) |

Table 1.3 Retention – waves 1-3. Proportions are out of all who consented and have any baseline data (ITT population), reported in % with 95% confidence interval.

|  |  | | Randomised intervention | | | |
| --- | --- | --- | --- | --- | --- | --- |
|  | All | | PInC | | IY | |
|  | N | Proportion | N | Proportion | N | Proportion |
| Randomised | nn |  | nn |  | Nn |  |
| Provide any follow-up data | nn | xx.x (xx.x – xx.x) | nn | xx.x (xx.x – xx.x) | nn | xx.x (xx.x – xx.x) |
| Provide follow-up data for |  |  |  |  |  |  |
| PSOC | nn | xx.x (xx.x – xx.x) | nn | xx.x (xx.x – xx.x) | nn | xx.x (xx.x – xx.x) |
| ECBI problem score | nn | xx.x (xx.x – xx.x) | nn | xx.x (xx.x – xx.x) | nn | xx.x (xx.x – xx.x) |
| ECBI intensity score | nn | xx.x (xx.x – xx.x) | nn | xx.x (xx.x – xx.x) | nn | xx.x (xx.x – xx.x) |
| SDQ | nn | xx.x (xx.x – xx.x) | nn | xx.x (xx.x – xx.x) | nn | xx.x (xx.x – xx.x) |
| GHQ | nn | xx.x (xx.x – xx.x) | nn | xx.x (xx.x – xx.x) | nn | xx.x (xx.x – xx.x) |
| ASRS (parent) Part A | nn | xx.x (xx.x – xx.x) | nn | xx.x (xx.x – xx.x) | nn | xx.x (xx.x – xx.x) |
| ASRS (parent) Part B | nn | xx.x (xx.x – xx.x) | nn | xx.x (xx.x – xx.x) | nn | xx.x (xx.x – xx.x) |
| PDHS challenging behaviour intensity | nn | xx.x (xx.x – xx.x) | nn | xx.x (xx.x – xx.x) | nn | xx.x (xx.x – xx.x) |
| PDHS challenging behaviour frequency | nn | xx.x (xx.x – xx.x) | nn | xx.x (xx.x – xx.x) | nn | xx.x (xx.x – xx.x) |
| PDHS parenting tasks intensity | nn | xx.x (xx.x – xx.x) | nn | xx.x (xx.x – xx.x) | nn | xx.x (xx.x – xx.x) |
| PDHS parenting tasks frequency | nn | xx.x (xx.x – xx.x) | nn | xx.x (xx.x – xx.x) | nn | xx.x (xx.x – xx.x) |
| CORC-GBO | nn | xx.x (xx.x – xx.x) | nn | xx.x (xx.x – xx.x) | nn | xx.x (xx.x – xx.x) |
| EQ-5D-5L | nn | xx.x (xx.x – xx.x) | nn | xx.x (xx.x – xx.x) | nn | xx.x (xx.x – xx.x) |
| PedsQL psychosocial health summary score | nn | xx.x (xx.x – xx.x) | nn | xx.x (xx.x – xx.x) | nn | xx.x (xx.x – xx.x) |
| PedsQL physical health summary score | nn | xx.x (xx.x – xx.x) | nn | xx.x (xx.x – xx.x) | nn | xx.x (xx.x – xx.x) |
| PedsQL total score | nn | xx.x (xx.x – xx.x) | nn | xx.x (xx.x – xx.x) | nn | xx.x (xx.x – xx.x) |

Table 1.4 Retention – wave 4. Proportions are out of all who consented and have any baseline data (ITT population), reported in % with 95% confidence interval.

|  |  | | Randomised intervention | | | |
| --- | --- | --- | --- | --- | --- | --- |
|  | All | | PInC | | IY | |
|  | N | Proportion | N | Proportion | N | Proportion |
| Randomised | nn |  | nn |  | Nn |  |
| Provide any follow-up data | nn | xx.x (xx.x – xx.x) | nn | xx.x (xx.x – xx.x) | nn | xx.x (xx.x – xx.x) |
| Provide follow-up data for |  |  |  |  |  |  |
| PSOC | nn | xx.x (xx.x – xx.x) | nn | xx.x (xx.x – xx.x) | nn | xx.x (xx.x – xx.x) |
| ECBI problem score | nn | xx.x (xx.x – xx.x) | nn | xx.x (xx.x – xx.x) | nn | xx.x (xx.x – xx.x) |
| ECBI intensity score | nn | xx.x (xx.x – xx.x) | nn | xx.x (xx.x – xx.x) | nn | xx.x (xx.x – xx.x) |
| SDQ | nn | xx.x (xx.x – xx.x) | nn | xx.x (xx.x – xx.x) | nn | xx.x (xx.x – xx.x) |
| GHQ | nn | xx.x (xx.x – xx.x) | nn | xx.x (xx.x – xx.x) | nn | xx.x (xx.x – xx.x) |
| ASRS (parent) Part A | nn | xx.x (xx.x – xx.x) | nn | xx.x (xx.x – xx.x) | nn | xx.x (xx.x – xx.x) |
| ASRS (parent) Part A | nn | xx.x (xx.x – xx.x) | nn | xx.x (xx.x – xx.x) | nn | xx.x (xx.x – xx.x) |
| PDHS challenging behaviour intensity | nn | xx.x (xx.x – xx.x) | nn | xx.x (xx.x – xx.x) | nn | xx.x (xx.x – xx.x) |
| PDHS challenging behaviour frequency | nn | xx.x (xx.x – xx.x) | nn | xx.x (xx.x – xx.x) | nn | xx.x (xx.x – xx.x) |
| PDHS parenting tasks intensity | nn | xx.x (xx.x – xx.x) | nn | xx.x (xx.x – xx.x) | nn | xx.x (xx.x – xx.x) |
| PDHS parenting tasks frequency | nn | xx.x (xx.x – xx.x) | nn | xx.x (xx.x – xx.x) | nn | xx.x (xx.x – xx.x) |
| CORC-GBO | nn | xx.x (xx.x – xx.x) | nn | xx.x (xx.x – xx.x) | nn | xx.x (xx.x – xx.x) |
| EQ-5D-5L | nn | xx.x (xx.x – xx.x) | nn | xx.x (xx.x – xx.x) | nn | xx.x (xx.x – xx.x) |
| PedsQL psychosocial health summary score | nn | xx.x (xx.x – xx.x) | nn | xx.x (xx.x – xx.x) | nn | xx.x (xx.x – xx.x) |
| PedsQL physical health summary score | nn | xx.x (xx.x – xx.x) | nn | xx.x (xx.x – xx.x) | nn | xx.x (xx.x – xx.x) |
| PedsQL total score | nn | xx.x (xx.x – xx.x) | nn | xx.x (xx.x – xx.x) | nn | xx.x (xx.x – xx.x) |

Table 2.1 Child baseline characteristics (ITT population)

|  |  |  | Randomised intervention | |
| --- | --- | --- | --- | --- |
|  |  | All | PInC | IY |
| Age (years) | N_OBS_ (N_MISS_)  Mean (SD)  Median (IQR)  [Range] | nn (nn)  xx.x (xx.x)  xx.x (xx.x, xx.x)  [xx.x, xx.x] | nn (nn)  xx.x (xx.x)  xx.x (xx.x, xx.x)  [xx.x, xx.x] | nn (nn)  xx.x (xx.x)  xx.x (xx.x, xx.x)  [xx.x, xx.x] |
| Gender | N_OBS_ (N_MISS_)  N(%) Male  N(%) Female | nn (nn)  nn (xx.x)  nn (xx.x) | nn (nn)  nn (xx.x)  nn (xx.x) | nn (nn)  nn (xx.x)  nn (xx.x) |
| Time since diagnosis | N_OBS_ (N_MISS_)  Mean (SD)  Median (IQR)  [Range] | nn (nn)  xx.x (xx.x)  xx.x (xx.x, xx.x)  [xx.x, xx.x] | nn (nn)  xx.x (xx.x)  xx.x (xx.x, xx.x)  [xx.x, xx.x] | nn (nn)  xx.x (xx.x)  xx.x (xx.x, xx.x)  [xx.x, xx.x] |
| Relationship with carer | N_OBS_ (N_MISS_)  N(%) Mother  N(%) Father  N(%) Grand Parent  N(%) Foster Parent  N(%) Other | nn (nn)  nn (xx.x)  nn (xx.x)  nn (xx.x)  nn (xx.x)  nn (xx.x) | nn (nn)  nn (xx.x)  nn (xx.x)  nn (xx.x)  nn (xx.x)  nn (xx.x) | nn (nn)  nn (xx.x)  nn (xx.x)  nn (xx.x)  nn (xx.x)  nn (xx.x) |
| On ADHD medication | N_OBS_ (N_MISS_)  N(%) No  N(%) Yes | nn (nn)  nn (xx.x)  nn (xx.x) | nn (nn)  nn (xx.x)  nn (xx.x) | nn (nn)  nn (xx.x)  nn (xx.x) |
| Time since starting medication | N_OBS_ (N_MISS_)  Mean (SD)  Median (IQR)  [Range] | nn (nn)  xx.x (xx.x)  xx.x (xx.x, xx.x)  [xx.x, xx.x] | nn (nn)  xx.x (xx.x)  xx.x (xx.x, xx.x)  [xx.x, xx.x] | nn (nn)  xx.x (xx.x)  xx.x (xx.x, xx.x)  [xx.x, xx.x] |
| Any other neurodevelopmental diagnoses | N_OBS_ (N_MISS_)  N(%) No  N(%) Yes | nn (nn)  nn (xx.x)  nn (xx.x) | nn (nn)  nn (xx.x)  nn (xx.x) | nn (nn)  nn (xx.x)  nn (xx.x) |

Table 2.2 Parent baseline characteristics (ITT population)

|  |  |  | Randomised intervention | |
| --- | --- | --- | --- | --- |
|  |  | All | PInC | IY |
| Age (years) | N_OBS_ (N_MISS_)  Mean (SD)  Median (IQR)  [Range] | nn (nn)  xx.x (xx.x)  xx.x (xx.x, xx.x)  [xx.x, xx.x] | nn (nn)  xx.x (xx.x)  xx.x (xx.x, xx.x)  [xx.x, xx.x] | nn (nn)  xx.x (xx.x)  xx.x (xx.x, xx.x)  [xx.x, xx.x] |
| Gender | N_OBS_ (N_MISS_)  N(%) Male  N(%) Female | nn (nn)  nn (xx.x)  nn (xx.x) | nn (nn)  nn (xx.x)  nn (xx.x) | nn (nn)  nn (xx.x)  nn (xx.x) |
| Ethnicity | N_OBS_ (N_MISS_)  N(%) White  N(%) Black  N(%) Asian  N(%) Chinese  N(%) Mixed  N(%) Prefer not to say | nn (nn)  nn (xx.x)  nn (xx.x)  nn (xx.x)  nn (xx.x)  nn (xx.x)  nn (xx.x) | nn (nn)  nn (xx.x)  nn (xx.x)  nn (xx.x)  nn (xx.x)  nn (xx.x)  nn (xx.x) | nn (nn)  nn (xx.x)  nn (xx.x)  nn (xx.x)  nn (xx.x)  nn (xx.x)  nn (xx.x) |
| Time since diagnosis | N_OBS_ (N_MISS_)  Mean (SD)  Median (IQR)  [Range] | nn (nn)  xx.x (xx.x)  xx.x (xx.x, xx.x)  [xx.x, xx.x] | nn (nn)  xx.x (xx.x)  xx.x (xx.x, xx.x)  [xx.x, xx.x] | nn (nn)  xx.x (xx.x)  xx.x (xx.x, xx.x)  [xx.x, xx.x] |
| Relationship with child | N_OBS_ (N_MISS_)  N(%) Mother  N(%) Father  N(%) Grand Parent  N(%) Foster Parent  N(%) Other | nn (nn)  nn (xx.x)  nn (xx.x)  nn (xx.x)  nn (xx.x)  nn (xx.x) | nn (nn)  nn (xx.x)  nn (xx.x)  nn (xx.x)  nn (xx.x)  nn (xx.x) | nn (nn)  nn (xx.x)  nn (xx.x)  nn (xx.x)  nn (xx.x)  nn (xx.x) |
| Level of deprivation (SIMD) | N_OBS_ (N_MISS_)  N(%) Q1  N(%) Q2  N(%) Q3  N(%) Q4  N(%) Q5 | nn (nn)  nn (xx.x)  nn (xx.x)  nn (xx.x)  nn (xx.x)  nn (xx.x) | nn (nn)  nn (xx.x)  nn (xx.x)  nn (xx.x)  nn (xx.x)  nn (xx.x) | nn (nn)  nn (xx.x)  nn (xx.x)  nn (xx.x)  nn (xx.x)  nn (xx.x) |
| Education | N_OBS_ (N_MISS_)  N(%) School leaving certificate, Access 1 or 2, National 1 or 2  Standard Grade, GCSE, O Level, …  … | nn (nn)  nn (xx.x)  nn (xx.x)  … | nn (nn)  nn (xx.x)  nn (xx.x)  … | nn (nn)  nn (xx.x)  nn (xx.x)  … |
| Average household income | N_OBS_ (N_MISS_)  N(%) 0 – 11,500  N(%) 11,501 - 33,500  N(%) 33,501 – 150,000  N(%) 150,001 and over | nn (nn)  nn (xx.x)  nn (xx.x)  nn (xx.x)  nn (xx.x) | nn (nn)  nn (xx.x)  nn (xx.x)  nn (xx.x)  nn (xx.x) | nn (nn)  nn (xx.x)  nn (xx.x)  nn (xx.x)  nn (xx.x) |

Table 3.1 Primary outcome - PSOC. ITT population.

|  |  |  | Randomised intervention | |
| --- | --- | --- | --- | --- |
|  |  | All | PInC | IY |
| Baseline | N_OBS_ (N_MISS_)  Mean (SD)  Median (IQR)  [Range] | nn (nn)  xx.x (xx.x)  xx.x (xx.x, xx.x)  [xx.x, xx.x] | nn (nn)  xx.x (xx.x)  xx.x (xx.x, xx.x)  [xx.x, xx.x] | nn (nn)  xx.x (xx.x)  xx.x (xx.x, xx.x)  [xx.x, xx.x] |
| Follow-up | N_OBS_ (N_MISS_)  Mean (SD)  Median (IQR)  [Range] | nn (nn)  xx.x (xx.x)  xx.x (xx.x, xx.x)  [xx.x, xx.x] | nn (nn)  xx.x (xx.x)  xx.x (xx.x, xx.x)  [xx.x, xx.x] | nn (nn)  xx.x (xx.x)  xx.x (xx.x, xx.x)  [xx.x, xx.x] |
| Participants with baseline and follow-up data only | | | | |
| N |  | nn | nn | Nn |
| Baseline | Mean (SD)  Median (IQR)  [Range] | xx.x (xx.x)  xx.x (xx.x, xx.x)  [xx.x, xx.x] | xx.x (xx.x)  xx.x (xx.x, xx.x)  [xx.x, xx.x] | xx.x (xx.x)  xx.x (xx.x, xx.x)  [xx.x, xx.x] |
| Follow-up | Mean (SD)  Median (IQR)  [Range] | xx.x (xx.x)  xx.x (xx.x, xx.x)  [xx.x, xx.x] | xx.x (xx.x)  xx.x (xx.x, xx.x)  [xx.x, xx.x] | xx.x (xx.x)  xx.x (xx.x, xx.x)  [xx.x, xx.x] |
| Change from baseline | Mean (SD)  Median (IQR)  [Range] | xx.x (xx.x)  xx.x (xx.x, xx.x)  [xx.x, xx.x] | xx.x (xx.x)  xx.x (xx.x, xx.x)  [xx.x, xx.x] | xx.x (xx.x)  xx.x (xx.x, xx.x)  [xx.x, xx.x] |
| Linear regression analysis predicting change from baseline from randomised intervention group adjusting for baseline PSOC, child age and gender | | | | |
|  |  | Adjusted mean difference (95% confidence interval) | | p-value |
| IY vs. PInC | | x.xx (x.xx, x.xx) | | x.xxxx |
| Residual standard deviation | | x.xx | |  |

Table 3.2 Primary outcome - PSOC. ITT population with 12-month follow-up.

|  |  |  | Randomised intervention | |
| --- | --- | --- | --- | --- |
|  |  | All | PInC | IY |
| Baseline | N_OBS_ (N_MISS_)  Mean (SD)  Median (IQR)  [Range] | nn (nn)  xx.x (xx.x)  xx.x (xx.x, xx.x)  [xx.x, xx.x] | nn (nn)  xx.x (xx.x)  xx.x (xx.x, xx.x)  [xx.x, xx.x] | nn (nn)  xx.x (xx.x)  xx.x (xx.x, xx.x)  [xx.x, xx.x] |
| Follow-up | N_OBS_ (N_MISS_)  Mean (SD)  Median (IQR)  [Range] | nn (nn)  xx.x (xx.x)  xx.x (xx.x, xx.x)  [xx.x, xx.x] | nn (nn)  xx.x (xx.x)  xx.x (xx.x, xx.x)  [xx.x, xx.x] | nn (nn)  xx.x (xx.x)  xx.x (xx.x, xx.x)  [xx.x, xx.x] |
| Participants with baseline and follow-up data only | | | | |
| N |  | nn | nn | nn |
| Baseline | Mean (SD)  Median (IQR)  [Range] | xx.x (xx.x)  xx.x (xx.x, xx.x)  [xx.x, xx.x] | xx.x (xx.x)  xx.x (xx.x, xx.x)  [xx.x, xx.x] | xx.x (xx.x)  xx.x (xx.x, xx.x)  [xx.x, xx.x] |
| Follow-up | Mean (SD)  Median (IQR)  [Range] | xx.x (xx.x)  xx.x (xx.x, xx.x)  [xx.x, xx.x] | xx.x (xx.x)  xx.x (xx.x, xx.x)  [xx.x, xx.x] | xx.x (xx.x)  xx.x (xx.x, xx.x)  [xx.x, xx.x] |
| Change from baseline | Mean (SD)  Median (IQR)  [Range] | xx.x (xx.x)  xx.x (xx.x, xx.x)  [xx.x, xx.x] | xx.x (xx.x)  xx.x (xx.x, xx.x)  [xx.x, xx.x] | xx.x (xx.x)  xx.x (xx.x, xx.x)  [xx.x, xx.x] |
| Linear regression analysis predicting change from baseline from randomised intervention group adjusting for baseline PSOC, child age and gender | | | | |
|  |  | Adjusted mean difference (95% confidence interval) | | p-value |
| IY vs. PInC | | x.xx (x.xx, x.xx) | | x.xxxx |
| Residual standard deviation | | x.xx | |  |

Table 3.3 Primary outcome - PSOC. PP population.

|  |  |  | Intervention received | |
| --- | --- | --- | --- | --- |
|  |  | All | PInC | IY |
| Baseline | N_OBS_ (N_MISS_)  Mean (SD)  Median (IQR)  [Range] | nn (nn)  xx.x (xx.x)  xx.x (xx.x, xx.x)  [xx.x, xx.x] | nn (nn)  xx.x (xx.x)  xx.x (xx.x, xx.x)  [xx.x, xx.x] | nn (nn)  xx.x (xx.x)  xx.x (xx.x, xx.x)  [xx.x, xx.x] |
| Follow-up | N_OBS_ (N_MISS_)  Mean (SD)  Median (IQR)  [Range] | nn (nn)  xx.x (xx.x)  xx.x (xx.x, xx.x)  [xx.x, xx.x] | nn (nn)  xx.x (xx.x)  xx.x (xx.x, xx.x)  [xx.x, xx.x] | nn (nn)  xx.x (xx.x)  xx.x (xx.x, xx.x)  [xx.x, xx.x] |
| Participants with baseline and follow-up data only | | | | |
| N |  | nn | nn | nn |
| Baseline | Mean (SD)  Median (IQR)  [Range] | xx.x (xx.x)  xx.x (xx.x, xx.x)  [xx.x, xx.x] | xx.x (xx.x)  xx.x (xx.x, xx.x)  [xx.x, xx.x] | xx.x (xx.x)  xx.x (xx.x, xx.x)  [xx.x, xx.x] |
| Follow-up | Mean (SD)  Median (IQR)  [Range] | xx.x (xx.x)  xx.x (xx.x, xx.x)  [xx.x, xx.x] | xx.x (xx.x)  xx.x (xx.x, xx.x)  [xx.x, xx.x] | xx.x (xx.x)  xx.x (xx.x, xx.x)  [xx.x, xx.x] |
| Change from baseline | Mean (SD)  Median (IQR)  [Range] | xx.x (xx.x)  xx.x (xx.x, xx.x)  [xx.x, xx.x] | xx.x (xx.x)  xx.x (xx.x, xx.x)  [xx.x, xx.x] | xx.x (xx.x)  xx.x (xx.x, xx.x)  [xx.x, xx.x] |
| Linear regression analysis predicting change from baseline from randomised intervention group adjusting for baseline PSOC, child age and gender | | | | |
|  |  | Adjusted mean difference (95% confidence interval) | | p-value |
| IY vs. PInC | | x.xx (x.xx, x.xx) | | x.xxxx |
| Residual standard deviation | | x.xx | |  |

Table 4.1.1 Secondary outcome – ECBI problem score. ITT population.

|  |  |  | Intervention received | |
| --- | --- | --- | --- | --- |
|  |  | All | PInC | IY |
| Baseline | N_OBS_ (N_MISS_)  N (%) ≤ 11  N (%) > 11 | nn (nn)  nn (xx.x)  nn (xx.x) | nn (nn)  nn (xx.x)  nn (xx.x) | nn (nn)  nn (xx.x)  nn (xx.x) |
| Follow-up | N_OBS_ (N_MISS_)  N (%) ≤ 11  N (%) > 11 | nn (nn)  nn (xx.x)  nn (xx.x) | nn (nn)  nn (xx.x)  nn (xx.x) | nn (nn)  nn (xx.x)  nn (xx.x) |
| Participants with baseline and follow-up data only | | | | |
| N |  | nn | nn | nn |
| Baseline | N_OBS_ (N_MISS_)  N (%) ≤ 11  N (%) > 11 | nn (nn)  nn (xx.x)  nn (xx.x) | nn (nn)  nn (xx.x)  nn (xx.x) | nn (nn)  nn (xx.x)  nn (xx.x) |
| Follow-up | N_OBS_ (N_MISS_)  N (%) ≤ 11  N (%) > 11 | nn (nn)  nn (xx.x)  nn (xx.x) | nn (nn)  nn (xx.x)  nn (xx.x) | nn (nn)  nn (xx.x)  nn (xx.x) |
| Logistic regression analysis predicting change from baseline from randomised intervention group adjusting for baseline ECBI problem score > 11, child age and gender | | | | |
|  |  | Adjusted odds ratio (95% confidence interval) | | p-value |
| IY vs. PInC | | x.xx (x.xx, x.xx) | | x.xxxx |

Table 4.1.2 Secondary outcome – ECBI problem score. ITT population with 12-month follow-up.

Table 4.2.1 Secondary outcome – ECBI intensity score. ITT population.

Table 4.2.2 Secondary outcome – ECBI intensity score. ITT population with 12-month follow-up.

Table 4.3.1 Secondary outcome - SDQ. ITT population.

Table 4.3.2 Secondary outcome - SDQ. ITT population with 12-month follow-up.

Table 4.3.3 Secondary outcome – SDQ using repeat baseline values where available. ITT population.

Table 4.4.1 Secondary outcome - GHQ. ITT population.

Table 4.4.2 Secondary outcome - GHQ. ITT population with 12-month follow-up.

Table 4.5.1 Secondary outcome – Parental ASRS Part A. ITT population.

Table 4.5.2 Secondary outcome – Parental ASRS Part A. ITT population with 12-month follow-up.

Table 4.6.1 Secondary outcome – Parental ASRS Part B. ITT population.

Table 4.6.2 Secondary outcome – Parental ASRS Part B. ITT population with 12-month follow-up.

Table 4.7.1 Secondary outcome – PDHS challenging behaviour intensity. ITT population.

Table 4.7.2 Secondary outcome - PDHS challenging behaviour intensity. ITT population with 12-month follow-up.

Table 4.7.3 Secondary outcome – PDHS challenging behaviour intensity using repeat baseline values where available. ITT population.

Table 4.8.1 Secondary outcome – PDHS challenging behaviour frequency. ITT population.

Table 4.8.2 Secondary outcome - PDHS challenging behaviour frequency. ITT population with 12-month follow-up.

Table 4.8.3 Secondary outcome – PDHS challenging behaviour frequency using repeat baseline values where available. ITT population.

Table 4.9.1 Secondary outcome – PDHS parenting tasks intensity. ITT population.

Table 4.9.2 Secondary outcome - PDHS parenting tasks intensity. ITT population with 12-month follow-up.

Table 4.9.3 Secondary outcome – PDHS parenting tasks intensity using repeat baseline values where available. ITT population.

Table 4.10.1 Secondary outcome – PDHS parenting tasks intensity. ITT population.

Table 4.10.2 Secondary outcome - PDHS parenting tasks intensity. ITT population with 12-month follow-up.

Table 4.10.3 Secondary outcome – PDHS parenting tasks intensity using repeat baseline values where available. ITT population.

Table 4.11.1 Secondary outcome – CORC-GBO. ITT population.

|  |  |  | Randomised intervention | |
| --- | --- | --- | --- | --- |
|  |  | All | PInC | IY |
| Baseline in goal which improved most | N_OBS_ (N_MISS_)  Mean (SD)  Median (IQR)  [Range] | nn (nn)  xx.x (xx.x)  xx.x (xx.x, xx.x)  [xx.x, xx.x] | nn (nn)  xx.x (xx.x)  xx.x (xx.x, xx.x)  [xx.x, xx.x] | nn (nn)  xx.x (xx.x)  xx.x (xx.x, xx.x)  [xx.x, xx.x] |
| Baseline in goal which improved least | N_OBS_ (N_MISS_)  Mean (SD)  Median (IQR)  [Range] | nn (nn)  xx.x (xx.x)  xx.x (xx.x, xx.x)  [xx.x, xx.x] | nn (nn)  xx.x (xx.x)  xx.x (xx.x, xx.x)  [xx.x, xx.x] | nn (nn)  xx.x (xx.x)  xx.x (xx.x, xx.x)  [xx.x, xx.x] |
| Average baseline across all goals | N_OBS_ (N_MISS_)  Mean (SD)  Median (IQR)  [Range] | nn (nn)  xx.x (xx.x)  xx.x (xx.x, xx.x)  [xx.x, xx.x] | nn (nn)  xx.x (xx.x)  xx.x (xx.x, xx.x)  [xx.x, xx.x] | nn (nn)  xx.x (xx.x)  xx.x (xx.x, xx.x)  [xx.x, xx.x] |
| Follow-up in goal which improved most | N_OBS_ (N_MISS_)  Mean (SD)  Median (IQR)  [Range] | nn (nn)  xx.x (xx.x)  xx.x (xx.x, xx.x)  [xx.x, xx.x] | nn (nn)  xx.x (xx.x)  xx.x (xx.x, xx.x)  [xx.x, xx.x] | nn (nn)  xx.x (xx.x)  xx.x (xx.x, xx.x)  [xx.x, xx.x] |
| Follow-up in goal which improved least | N_OBS_ (N_MISS_)  Mean (SD)  Median (IQR)  [Range] | nn (nn)  xx.x (xx.x)  xx.x (xx.x, xx.x)  [xx.x, xx.x] | nn (nn)  xx.x (xx.x)  xx.x (xx.x, xx.x)  [xx.x, xx.x] | nn (nn)  xx.x (xx.x)  xx.x (xx.x, xx.x)  [xx.x, xx.x] |
| Average follow-up across all goals | N_OBS_ (N_MISS_)  Mean (SD)  Median (IQR)  [Range] | nn (nn)  xx.x (xx.x)  xx.x (xx.x, xx.x)  [xx.x, xx.x] | nn (nn)  xx.x (xx.x)  xx.x (xx.x, xx.x)  [xx.x, xx.x] | nn (nn)  xx.x (xx.x)  xx.x (xx.x, xx.x)  [xx.x, xx.x] |
| Participants with baseline and follow-up data only | | | | |
| N |  | nn | nn | Nn |
| Baseline in goal which improved most | N_OBS_ (N_MISS_)  Mean (SD)  Median (IQR)  [Range] | nn (nn)  xx.x (xx.x)  xx.x (xx.x, xx.x)  [xx.x, xx.x] | nn (nn)  xx.x (xx.x)  xx.x (xx.x, xx.x)  [xx.x, xx.x] | nn (nn)  xx.x (xx.x)  xx.x (xx.x, xx.x)  [xx.x, xx.x] |
| Baseline in goal which improved least | N_OBS_ (N_MISS_)  Mean (SD)  Median (IQR)  [Range] | nn (nn)  xx.x (xx.x)  xx.x (xx.x, xx.x)  [xx.x, xx.x] | nn (nn)  xx.x (xx.x)  xx.x (xx.x, xx.x)  [xx.x, xx.x] | nn (nn)  xx.x (xx.x)  xx.x (xx.x, xx.x)  [xx.x, xx.x] |
| Average baseline across all goals | N_OBS_ (N_MISS_)  Mean (SD)  Median (IQR)  [Range] | nn (nn)  xx.x (xx.x)  xx.x (xx.x, xx.x)  [xx.x, xx.x] | nn (nn)  xx.x (xx.x)  xx.x (xx.x, xx.x)  [xx.x, xx.x] | nn (nn)  xx.x (xx.x)  xx.x (xx.x, xx.x)  [xx.x, xx.x] |
| Follow-up in goal which improved most | N_OBS_ (N_MISS_)  Mean (SD)  Median (IQR)  [Range] | nn (nn)  xx.x (xx.x)  xx.x (xx.x, xx.x)  [xx.x, xx.x] | nn (nn)  xx.x (xx.x)  xx.x (xx.x, xx.x)  [xx.x, xx.x] | nn (nn)  xx.x (xx.x)  xx.x (xx.x, xx.x)  [xx.x, xx.x] |
| Follow-up in goal which improved least | N_OBS_ (N_MISS_)  Mean (SD)  Median (IQR)  [Range] | nn (nn)  xx.x (xx.x)  xx.x (xx.x, xx.x)  [xx.x, xx.x] | nn (nn)  xx.x (xx.x)  xx.x (xx.x, xx.x)  [xx.x, xx.x] | nn (nn)  xx.x (xx.x)  xx.x (xx.x, xx.x)  [xx.x, xx.x] |
| Average follow-up across all goals | N_OBS_ (N_MISS_)  Mean (SD)  Median (IQR)  [Range] | nn (nn)  xx.x (xx.x)  xx.x (xx.x, xx.x)  [xx.x, xx.x] | nn (nn)  xx.x (xx.x)  xx.x (xx.x, xx.x)  [xx.x, xx.x] | nn (nn)  xx.x (xx.x)  xx.x (xx.x, xx.x)  [xx.x, xx.x] |
| Change from baseline in goal which improved most | Mean (SD)  Median (IQR)  [Range] | xx.x (xx.x)  xx.x (xx.x, xx.x)  [xx.x, xx.x] | xx.x (xx.x)  xx.x (xx.x, xx.x)  [xx.x, xx.x] | xx.x (xx.x)  xx.x (xx.x, xx.x)  [xx.x, xx.x] |
| Change from baseline in goal which improved least | Mean (SD)  Median (IQR)  [Range] | xx.x (xx.x)  xx.x (xx.x, xx.x)  [xx.x, xx.x] | xx.x (xx.x)  xx.x (xx.x, xx.x)  [xx.x, xx.x] | xx.x (xx.x)  xx.x (xx.x, xx.x)  [xx.x, xx.x] |
| Average change from baseline across all goals | Mean (SD)  Median (IQR)  [Range] | xx.x (xx.x)  xx.x (xx.x, xx.x)  [xx.x, xx.x] | xx.x (xx.x)  xx.x (xx.x, xx.x)  [xx.x, xx.x] | xx.x (xx.x)  xx.x (xx.x, xx.x)  [xx.x, xx.x] |

Table 4.11.2 Secondary outcome – CORC-GBO. ITT population with 12-month follow-up.

Table 4.12.1 Secondary outcome – EQ-5D. ITT population.

Table 4.12.2 Secondary outcome – EQ-5D. ITT population with 12-month follow-up.

Table 4.13.1 Secondary outcome - PedsQL psychosocial health summary score. ITT population.

Table 4.13.2 Secondary outcome - PedsQL psychosocial health summary score. ITT population with 12-month follow-up.

Table 4.14.1 Secondary outcome - PedsQL physical health summary score. ITT population.

Table 4.14.2 Secondary outcome - PedsQL physical health summary score. ITT population with 12-month follow-up.

Table 4.15.1 Secondary outcome - PedsQL total score. ITT population.

Table 4.15.2 Secondary outcome - PedsQL total score. ITT population with 12-month follow-up.

Table 5.1 Attendance at group sessions

|  |  |  | Randomised intervention | |
| --- | --- | --- | --- | --- |
|  |  | All | PInC | IY |
| Proportion of sessions attended (%) | N_OBS_ (N_MISS_)  Mean (SD)  Median (IQR)  [Range] | nn (nn)  xx.x (xx.x)  xx.x (xx.x, xx.x)  [xx.x, xx.x] | nn (nn)  xx.x (xx.x)  xx.x (xx.x, xx.x)  [xx.x, xx.x] | nn (nn)  xx.x (xx.x)  xx.x (xx.x, xx.x)  [xx.x, xx.x] |

Table 5.2 Attendance at group sessions by sessions – PinC

| Session | Number not withdrawn | Number attended | Percentage attended |
| --- | --- | --- | --- |
| Week 1 | nn | nn | xx.x |
| Week 2 | nn | nn | xx.x |
| Week 3 | nn | nn | xx.x |
| Week 4 | nn | nn | xx.x |
| Week 5 | nn | nn | xx.x |
| Follow-up | nn | nn | xx.x |
| Attended at least 2 sessions | nn | nn | xx.x |

Table 5.3 Attendance at group sessions by sessions – IY

| Session | Number not withdrawn | Number attended | Percentage attended |
| --- | --- | --- | --- |
| Week 1 | nn | nn | xx.x |
| Week 2 | nn | nn | xx.x |
| Week 3 | nn | Nn | xx.x |
| Week 4 | nn | Nn | xx.x |
| Week 5 | nn | Nn | xx.x |
| … | … | … | … |
| Week 14 | nn | Nn | xx.x |
| Attended at least 7 sessions | nn | Nn | xx.x |

Table 6.1 Withdrawals from the intervention

|  |  |  | Randomised intervention | |
| --- | --- | --- | --- | --- |
|  |  | All | PInC | IY |
| Withdrawals | N_OBS_ (N_MISS_)  N(%) No  N(%) Yes | nn (nn)  nn (xx.x)  nn (xx.x) | nn (nn)  nn (xx.x)  nn (xx.x) | nn (nn)  nn (xx.x)  nn (xx.x) |
| Withdrawal reasons | N_OBS_ (N_MISS_)  N(%) Wished to attend another intervention  N(%) Didn’t want to continue  N(%) Couldn’t attend due to ill health  N(%) time commitment  N(%) No reason given | nn (nn)  nn (xx.x)  nn (xx.x) | nn (nn)  nn (xx.x)  nn (xx.x) | nn (nn)  nn (xx.x)  nn (xx.x) |

Table 6.2 Withdrawals from the study

|  |  |  | Randomised intervention | |
| --- | --- | --- | --- | --- |
|  |  | All | PInC | IY |
| Withdrawals | N_OBS_ (N_MISS_)  N(%) No  N(%) Yes | nn (nn)  nn (xx.x)  nn (xx.x) | nn (nn)  nn (xx.x)  nn (xx.x) | nn (nn)  nn (xx.x)  nn (xx.x) |
| Withdrawal reasons | N_OBS_ (N_MISS_)  N(%) Unhappy with baseline questionnaire  N(%) Too busy  N(%) Did not attend baseline assessment… | nn (nn)  nn (xx.x)  nn (xx.x)  nn (xx.x) | nn (nn)  nn (xx.x)  nn (xx.x)  nn (xx.x) | nn (nn)  nn (xx.x)  nn (xx.x)  nn (xx.x) |

Table 7 Summary of adverse events

|  |  |  | Randomised intervention | |
| --- | --- | --- | --- | --- |
|  |  | All | PInC | IY |
| Participants experiencing at least one SAE | N(%) | nn (xx.x) | nn (xx.x) | nn (xx.x) |
| Number of events | N | nn | nn | nn |
| Required hospitalisation | N(%) | nn (xx.x) | nn (xx.x) | nn (xx.x) |
| … | … | … | … | … |
| Severity | N_OBS_ (N_MISS_)  N(%) Mild  N(%) Moderate  N(%) Severe | nn (nn)  nn (xx.x)  nn (xx.x)  nn (xx.x) | nn (nn)  nn (xx.x)  nn (xx.x)  nn (xx.x) | nn (nn)  nn (xx.x)  nn (xx.x)  nn (xx.x) |
| Expectedness | N_OBS_ (N_MISS_)  N(%) Expected  N(%) Unexpected | nn (nn)  nn (xx.x)  nn (xx.x) | nn (nn)  nn (xx.x)  nn (xx.x) | nn (nn)  nn (xx.x)  nn (xx.x) |
| Relation to intervention | N_OBS_ (N_MISS_)  N(%) Related  N(%) Unrelated | nn (nn)  nn (xx.x)  nn (xx.x) | nn (nn)  nn (xx.x)  nn (xx.x) | nn (nn)  nn (xx.x)  nn (xx.x) |
| Outcome | N_OBS_ (N_MISS_)  N(%) Recovered  N(%) Recovered with sequelae  N(%) Recovering  N(%) Not recovered  N(%) Fatal | nn (nn)  nn (xx.x)  nn (xx.x)  nn (xx.x)  nn (xx.x)  nn (xx.x) | nn (nn)  nn (xx.x)  nn (xx.x)  nn (xx.x)  nn (xx.x)  nn (xx.x) | nn (nn)  nn (xx.x)  nn (xx.x)  nn (xx.x)  nn (xx.x)  nn (xx.x) |
| Duration (days) | N_OBS_ (N_MISS_)  Mean (SD)  Median (IQR)  [Range] | nn (nn)  xx.x (xx.x)  xx.x (xx.x, xx.x)  [xx.x, xx.x] | nn (nn)  xx.x (xx.x)  xx.x (xx.x, xx.x)  [xx.x, xx.x] | nn (nn)  xx.x (xx.x)  xx.x (xx.x, xx.x)  [xx.x, xx.x] |

Table 8 Medications

|  |  |  | Randomised intervention | |
| --- | --- | --- | --- | --- |
|  |  | All | PInC | IY |
| Medication 1 | N(%) | nn (xx.x) | nn (xx.x) | nn (xx.x) |
| … |  |  |  |  |
