# Supplementary material for: Families with neurodevelopmental diagnoses are not ‘Hard to Reach’: Findings from a feasibility trial comparing parenting programmes for parents of children with ADHD
Source: PLoS One. 2025 Sep 17;20(9):e0323959. doi: 10.1371/journal.pone.0323959 (PMC12443243; doi:10.1371/journal.pone.0323959)
Supplement: Attachment — Submitted filename: PONE-D-24-36397_reviewer.pdf [file pone.0323959.s003.pdf]

PLOS ONE

Families with neurodevelopmental diagnoses are not 'Hard to Reach': Findings from a feasibility trial comparing parenting programmes for parents of children with ADHD

--Manuscript Draft--

**Manuscript Number:**	PONE-D-24-36397
**Article Type:**	Research Article
**Full Title:**	Families with neurodevelopmental diagnoses are not 'Hard to Reach': Findings from a feasibility trial comparing parenting programmes for parents of children with ADHD
**Short Title:**	Families with neurodevelopmental diagnoses are not 'Hard to Reach'
**Corresponding Author:**	Lucy Thompson<br>University of Glasgow School of Health and Wellbeing<br>UNITED KINGDOM OF GREAT BRITAIN AND NORTHERN IRELAND
**Keywords:**	ADHD; attention-deficit/hyperactivity disorder; Parenting; parent support; parent education; feasibility trial
**Abstract:**	Attention-Deficit/Hyperactivity Disorder (ADHD) is defined by impairing levels of inattention and/or hyperactivity-impulsivity. It occurs in at least 5% of school age children and 2.5% of adults worldwide, and is associated with multiple negative outcomes throughout life. There is good evidence to support the efficacy of pharmacological treatment of individuals affected, and also of behavioural training for parents of children with ADHD, though the optimal focus and format to support change has yet to be established. This feasibility, parallel-group, randomised, controlled, pilot trial examined the feasibility of comparing two parenting programmes for families of school-aged children with ADHD. Parents of children aged 5-12 years with a clinical diagnosis of ADHD, referred to a regional integrated ADHD pathway, were randomly allocated (1:1 in permuted blocks of 4) either to a Parents InC (PInC) or Incredible Years (IY) parenting group. The primary feasibility outcomes were recruitment and retention rates. The putative primary effectiveness outcome measure was Parenting Sense of Competence Scale (PSOC) and secondary outcome measures included the behavioural, physical, and emotional functioning of the child as well as health and ADHD symptoms of the parents at 12 (or 6 in final four participants) months post-randomisation. Process and economic evaluations were also included. We recruited 30/52 (58%) eligible participants (14 randomised to PInC, 16 to IY). Twenty-two participants (73%) provided follow-up quantitative data and 24 (80%) participated in qualitative interviews. PSOC scores were suggestive of greater improvement in PInC groups than IY groups. Power calculations suggest we will need to recruit 230 participants for a definitive RCT. Health economic analysis showed PInC had a lower per-group costs. Participant feedback on research procedures and methods was positive, and interviews and process evaluation provided a rich source of learning points to take forward into a future trial.
**Order of Authors:**	Fiona Sim
	Lindsay Dalgarno
	Emma McIntosh
	Caroline Haig
	Patrycja Duklas
	Alex McConnachie
	Christopher Gillberg
	Helen Minnis
	Lucy Thompson
**Additional Information:**	
**Question**	**Response**

Powered by Editorial Manager® and ProduXion Manager® from Aries Systems Corporation

**Financial Disclosure**<br><br>Enter a financial disclosure statement that describes the sources of funding for the work included in this submission. Review the submission guidelines for detailed requirements. View published research articles from *PLOS ONE* for specific examples.<br><br>This statement is required for submission and **will appear in the published article** if the submission is accepted. Please make sure it is accurate.<br><br>**Funded studies**<br>Enter a statement with the following details:<br>• Initials of the authors who received each award<br>• Grant numbers awarded to each author<br>• The full name of each funder<br>• URL of each funder website<br>• Did the sponsors or funders play any role in the study design, data collection and analysis, decision to publish, or preparation of the manuscript?<br><br>Did you receive funding for this work?	Yes
Please add funding details.<br>as follow-up to "**Financial Disclosure**<br><br>Enter a financial disclosure statement that describes the sources of funding for the work included in this submission. Review the submission guidelines for detailed requirements. View published research articles from *PLOS ONE* for specific examples.<br><br>This statement is required for submission and **will appear in the published article** if the submission is accepted. Please make sure it is accurate.	This work was funded by a research grant from the Scottish Government Health Directorates' Chief Scientist Office (https://www.cso.scot.nhs.uk/), ref: HIPS/17/58. The funder had no direct involvement in the design, data collection, analysis, decision to publish, or preparation in the manuscript.

**Funded studies**<br>Enter a statement with the following details:<br>• Initials of the authors who received each award<br>• Grant numbers awarded to each author<br>• The full name of each funder<br>• URL of each funder website<br>• Did the sponsors or funders play any role in the study design, data collection and analysis, decision to publish, or preparation of the manuscript?<br><br>Did you receive funding for this work?"	
Please select the country of your main research funder (please select carefully as in some cases this is used in fee calculation).<br>as follow-up to "**Financial Disclosure**<br><br>Enter a financial disclosure statement that describes the sources of funding for the work included in this submission. Review the submission guidelines for detailed requirements. View published research articles from *PLOS ONE* for specific examples.<br><br>This statement is required for submission and **will appear in the published article** if the submission is accepted. Please make sure it is accurate.<br><br>**Funded studies**<br>Enter a statement with the following details:<br>• Initials of the authors who received each award<br>• Grant numbers awarded to each author<br>• The full name of each funder<br>• URL of each funder website<br>• Did the sponsors or funders play any role in the study design, data collection and analysis, decision to publish, or preparation of the manuscript?<br><br>Did you receive funding for this work?"	UNITED KINGDOM - GB
**Competing Interests**	The authors have declared that no competing interests exist.

Use the instructions below to enter a competing interest statement for this submission. On behalf of all authors, disclose any competing interests that could be perceived to bias this work—acknowledging all financial support and any other relevant financial or non-financial competing interests.<br><br>This statement is required for submission and **will appear in the published article** if the submission is accepted. Please make sure it is accurate and that any funding sources listed in your Funding Information later in the submission form are also declared in your Financial Disclosure statement.<br><br>View published research articles from *PLOS ONE* for specific examples.<br><br>**NO authors have competing interests**<br><br>Enter: *The authors have declared that no competing interests exist.*<br><br>**Authors with competing interests**<br><br>Enter competing interest details beginning with this statement:<br><br>*I have read the journal's policy and the authors of this manuscript have the following competing interests: [insert competing interests here]*<br><br>* typeset	
**Ethics Statement**<br><br>Enter an ethics statement for this submission. This statement is required if the study involved:<br><br>• Human participants<br>• Human specimens or tissue<br>• Vertebrate animals or cephalopods<br>• Vertebrate embryos or tissues	This study was reviewed and approved by the NHS North of Scotland Research Ethics Service on 13 December 2018, reference 18/NS/0124. Written informed consent was obtained for all participants in the research.

Powered by Editorial Manager® and ProduXion Manager® from Aries Systems Corporation

• Field research<br><br>Write "N/A" if the submission does not require an ethics statement.<br><br>General guidance is provided below. Consult the submission guidelines for detailed instructions. **Make sure that all information entered here is included in the Methods section of the manuscript.**	

Powered by Editorial Manager® and ProduXion Manager® from Aries Systems Corporation

**Format for specific study types**<br><br>**Human Subject Research (involving human participants and/or tissue)**<br>• Give the name of the institutional review board or ethics committee that approved the study<br>• Include the approval number and/or a statement indicating approval of this research<br>• Indicate the form of consent obtained (written/oral) or the reason that consent was not obtained (e.g. the data were analyzed anonymously)<br><br>**Animal Research (involving vertebrate animals, embryos or tissues)**<br>• Provide the name of the Institutional Animal Care and Use Committee (IACUC) or other relevant ethics board that reviewed the study protocol, and indicate whether they approved this research or granted a formal waiver of ethical approval<br>• Include an approval number if one was obtained<br>• If the study involved *non-human primates*, add *additional details* about animal welfare and steps taken to ameliorate suffering<br>• If anesthesia, euthanasia, or any kind of animal sacrifice is part of the study, include briefly which substances and/or methods were applied<br><br>**Field Research**<br><br>Include the following details if this study involves the collection of plant, animal, or other materials from a natural setting:<br>• Field permit number<br>• Name of the institution or relevant body that granted permission	
**Data Availability**<br><br>Authors are required to make all data underlying the findings described fully available, without restriction, and from the time of publication. PLOS allows rare exceptions to address legal and ethical concerns. See the PLOS Data Policy and FAQ for detailed information.	No - some restrictions will apply

Powered by Editorial Manager® and ProduXion Manager® from Aries Systems Corporation

A Data Availability Statement describing where the data can be found is required at submission. Your answers to this question constitute the Data Availability Statement and **will be published in the article**, if accepted.<br><br>**Important:** Stating 'data available on request from the author' is not sufficient. If your data are only available upon request, select 'No' for the first question and explain your exceptional situation in the text box.<br><br>Do the authors confirm that all data underlying the findings described in their manuscript are fully available without restriction?	
**Describe where the data may be found in full sentences. If you are copying our sample text, replace any instances of XXX with the appropriate details.**<br><br>• If the data are **held or will be held in a public repository**, include URLs, accession numbers or DOIs. If this information will only be available after acceptance, indicate this by ticking the box below. For example: *All XXX files are available from the XXX database (accession number(s) XXX, XXX.*).<br>• If the data are all contained **within the manuscript and/or Supporting Information files**, enter the following: *All relevant data are within the manuscript and its Supporting Information files.*<br>• If neither of these applies but you are able to provide **details of access elsewhere**, with or without limitations, please do so. For example:<br><br>*Data cannot be shared publicly because of [XXX]. Data are available from the XXX Institutional Data Access / Ethics Committee (contact via XXX) for researchers who meet the criteria for access to confidential data.*<br><br>*The data underlying the results presented in the study are available from (include the name of the third party*	We intend to make an anonymised dataset available in a public repository after acceptance for publication.

and contact information or URL).<br>• This text is appropriate if the data are owned by a third party and authors do not have permission to share the data.<br><br>* typeset	
Additional data availability information:	Tick here if the URLs/accession numbers/DOIs will be available only after acceptance of the manuscript for publication so that we can ensure their inclusion before publication.

Powered by Editorial Manager® and ProduXion Manager® from Aries Systems Corporation

Manuscript

Click here to access/download;Manuscript;SPARCLAR PLOSOne manuscript 27Jul24.docx

Families with neurodevelopmental diagnoses are not ‘Hard to Reach’: Findings from a feasibility trial comparing parenting programmes for parents of children with ADHD

Fiona Sim[1], Lindsay Dalgarno[1], Emma McIntosh[1], Caroline Haig[2], Patrycja Duklas[2], Alex McConnachie[2], Christopher Gillberg[1,4], Helen Minnis[1,4¶], Lucy Thompson[1,3,4*¶]

[1] School of Health and Wellbeing, University of Glasgow, Glasgow, Scotland

[2] Robertson Centre for Biostatistics, School of Health and Wellbeing, University of Glasgow, Glasgow, Scotland

[3] Institute of Applied Health Sciences, University of Aberdeen, Inverness, Scotland

[4] Gillberg Neuropsychiatry Centre, University of Gothenburg, Gothenburg, Sweden

* Corresponding author: Lucy.Thompson@abdn.ac.uk

¶ Denotes joint senior authorship

ABSTRACT

Attention-Deficit/Hyperactivity Disorder (ADHD) is defined by impairing levels of inattention and/or hyperactivity-impulsivity. It occurs in at least 5% of school age children and 2.5% of adults worldwide, and is associated with multiple negative outcomes throughout life. There is good evidence to support the efficacy of pharmacological treatment of individuals affected, and also of behavioural training for parents of children with ADHD, though the optimal focus and format to support change has yet to be established. This feasibility, parallel-group, randomised, controlled, pilot trial examined the feasibility of comparing two parenting programmes for families of school-aged children with ADHD. Parents of children aged 5-12 years with a clinical diagnosis of ADHD, referred to a regional integrated ADHD pathway, were randomly allocated (1:1 in permuted blocks of 4) either to a Parents InC (PInC) or Incredible Years (IY) parenting group. The primary feasibility outcomes were recruitment and retention rates. The putative primary effectiveness outcome measure was Parenting Sense of Competence Scale (PSOC) and secondary outcome measures included the behavioural, physical, and emotional functioning of the child as well as health and ADHD symptoms of the parents at 12 (or 6 in final four participants) months post-randomisation. Process and economic evaluations were also included. We recruited 30/52 (58%) eligible participants (14 randomised to PInC, 16 to IY). Twenty-two participants (73%) provided follow-up quantitative data and 24 (80%) participated in qualitative interviews. PSOC scores were suggestive of greater improvement in PInC groups than IY groups. Power calculations suggest we will need to recruit 230 participants for a definitive RCT. Health economic analysis showed PInC had a lower per-group costs. Participant feedback on research procedures and methods was positive, and interviews and process evaluation provided a rich source of learning points to take forward into a future trial.

Trial registration:

Clinical Trials, protocol registration system: NCT03832270.

INTRODUCTION

Attention-Deficit/Hyperactivity Disorder (ADHD) is defined by impairing levels of inattention and/or hyperactivity-impulsivity (1) and demonstrates a high level of familial occurrence. ADHD is associated with multiple negative outcomes throughout life (2, 3). Prevalence estimates suggest ADHD occurs in approximately 5% of school age children and 2.5% of adults worldwide (4). In childhood it can manifest in difficulty organising tasks and sustaining attention during schoolwork or play, frequently losing belongings and an inability to stay seated to a degree inconsistent with age or developmental level (1, 5). ADHD displays a high level of symptomatic overlap with other neurodevelopmental diagnoses including developmental coordination disorder, language disorder, autism and intellectual disability (6). Externalising disorders such as oppositional defiant disorder and conduct disorder show a particularly high level of co-occurrence with ADHD in childhood (1) presenting challenges, not only to the individual experiencing the symptoms, but also to those responsible for their care and education (5, 7).

Many parents manage well when the child has ADHD, and there is evidence that maternal ADHD can enhance the care-giving relationship (8). However, challenges in the care-giving relationship resulting from ADHD symptoms can express themselves in negative communication styles, increased permissiveness and over-reactivity and lower responsivity than in families of children without ADHD (9-13). Parenting a child with ADHD can be stressful and has been associated with child maltreatment (14). There is also evidence that relational challenges can be compounded when parents exhibit ADHD traits themselves, which can result in consequences for both the child's quality of life and management of the child's ADHD symptoms (15-19). Current best practice recommendations for the treatment of ADHD in children outline a three tiered approach of ADHD-focussed parenting groups as a first-line treatment following diagnosis, pharmacotherapy in children aged 6 years and older, and Cognitive Behavioural Therapy (20). The efficacy of behavioural training for parents of children with ADHD symptoms is well established in the general population (21, 22), though there is some indication

that the presence of parental ADHD symptoms may mediate the efficacy of parent training for children with ADHD (23, 24).

Factors which determine the efficacy of a parenting intervention, such as attendance, engagement and subsequent consistent application of learning, present real challenges to parents particularly when they themselves have ADHD symptoms: difficulty with organisation and with conforming to routine are likely to be fundamental (25). Low recruitment and retention to parenting programmes have significant impacts on their effectiveness (26) and barriers such as parental psychopathology (27, 28), stigma, childcare and programme/service barriers have been identified as key (29). These barriers can appear almost insurmountable for families who, for personal (e.g. education, psychopathology) and situational (e.g. deprivation, homelessness) circumstances, consistently struggle to access or engage in services (30). If services are to be accessible and inclusive, then clinical research regarding effectiveness must ensure that marginalised families are able to participate.

This article presents findings from a feasibility randomised controlled trial (RCT) comparing two parenting interventions for parents of children with an ADHD diagnosis. The interventions were: Parents In Control (Parents InC) (31), designed specifically for parents of children with an ADHD diagnosis with a particular focus on parental sense of self competence; and the Incredible Years (IY) (32), a group parenting support intervention aimed at strengthening parent-child interactions and attachment, reducing harsh discipline and fostering parents' ability to promote children's social, emotional, and academic development. IY is established as an effective intervention programme for parents of children with disruptive behaviour problems, with positive outcomes in child behaviour, parent self-efficacy, and parent wellbeing (21, 33, 34). Parents InC is delivered over six sessions to parents of children with a diagnosis of ADHD and IY is delivered over fourteen sessions to parents of children with a range of behavioural concerns. Preliminary evaluation of the programme has been positive, with some evidence of improvement in parent-reported frequency and intensity of child

problem behaviours in a pre-post study (35). At the time of conducting this research, Parents InC is the first-line parenting programme for parents of a child with a diagnosis of ADHD in NHS Fife.

The aims of this study were to identify whether a) parents of children recently diagnosed with ADHD were willing to be randomised to either Parents InC or Incredible Years, and b) sufficient numbers of families could be recruited and retained such that a full-scale randomised controlled trial is likely to be feasible. The following secondary research questions were addressed, to inform future definitive trial design, and will be reported here:

- Are research procedures and efficacy measures feasible and acceptable to participating families?
- Do families participating in Parents InC achieve similar scores on the parenting sense of competence scale at 12 months post randomisation as those in the comparison arm (IY)?
- Do the two intervention arms significantly differ on any other measures?
- What is the mean cost per participant of Parents InC?

MATERIALS AND METHODS

Trial Design

We conducted a feasibility parallel-group RCT pilot study, comparing two parenting programmes for families of school-aged children with ADHD. An equal allocation ratio of 1:1 was adopted for this trial in order to provide the greatest power for testing feasibility of a future definitive RCT.

Participants

Parents of 5–12-year-old children with a formal diagnosis of ADHD were eligible for inclusion. The children had had a standardised assessment from a paediatrician or a psychiatrist after referral to the Fife integrated ADHD pathway between January 2019 and January 2020**.**

Families attending other parenting group-based interventions were excluded, as were parents who had low proficiency in English (as this could compromise ability to complete research measures or

participate in a group intervention). Participants already taking part in research on a parenting intervention were also ineligible to participate.

Target recruitment figures were based on known clinical throughput within the Fife integrated ADHD pathway and on known recruitment and retention rates from a similar intervention trial conducted in a more socioeconomically deprived population (36). In order to obtain follow-up data on at least 40 participants (20 per arm), we expected to need to approach 100 families in total (assuming a 60% response-rate, i.e., n=60 randomised, 30 per arm; and two thirds of participating families being available for follow-up).

Procedure

All participants were recruited within the NHS Fife area between 1st January 2019 and 31st January 2020. Those eligible to participate, who were part of the Fife integrated ADHD pathway, were informed about the study through one of three channels: 1) during a routine appointment with referring paediatricians and psychiatrists; 2) an assistant clinical psychologist within the ADHD pathway telephoned families on the waiting list for Parents InC groups; or 3) An invitation to participate in Parents InC groups containing a tear-off 'Expression of Interest [research]' form was sent to the parents' home address by the clinical psychology team; eligible participants who returned this form were then contacted directly by the trial team. Referrers were provided with eligibility criteria to ensure appropriate referrals to the research trial. Eligibility was confirmed by the research team on receipt of the referral information.

A participant information sheet and consent form were then posted to the home of potential participants and followed up with a telephone call within one week of the information being posted to allow informed written consent to be obtained. During this call, participants were asked to return the freepost consent form and an appointment was made to collect baseline data. Participants were given the choice of their preferred location for data collection, and the options were as follows: 1)

face-to-face interview at the participant's home; 2) face-to-face interview in a clinical assessment room at an NHS facility in Fife; or 3) remotely via telephone or video call. Baseline data collection took 60-90mins, after which participants were randomised using an Interactive Voice Response System at the clinical research facility. Participants were allocated 1:1 (in permuted blocks of 4 with no stratification or minimisation) to either PInC or IY, and referral information was emailed to the relevant intervention team by the clinical research facility staff. A teacher-report copy of the Strengths & Difficulties Questionnaire (SDQ) was left with the parent/carer at the baseline visit for the parent to pass on to the child's class teacher; these were returned to the trial office by the class teacher via freepost envelope.

Two separate 3-month iterations of recruitment / intervention were planned to allow time for reflection and refinement for each iteration. A third round of intervention was planned for any remaining participants who were unable to take up participation in the first group they were offered. A subsequent decision was taken to include a fourth iteration in response to referral delays. Participants in the first three recruitment iterations received follow-up data collection 12 months post-randomisation. Those participants in the fourth iteration received theirs at 6 months post randomisation. In response to the nationally enforced social distancing measures brought into force on the 23rd March 2020 during the international COVID19 pandemic, the trial moved entirely to remote data collection and all follow-up data was collected via telephone or video call.

Qualitative methods

Semi-structured interviews were conducted with consenting trial participants at baseline and 12 months post-randomisation, and interviews held with intervention practitioners during and after the intervention.

Qualitative methods were employed to explore the following areas:

- Acceptability of research procedures to participating families;

- Quality of life for families living with ADHD;
- Parental expectations and experiences of intervention;
- Acceptability of research procedures to participating practitioners.

Measures

The *primary intervention efficacy outcome*, for a future definitive RCT is likely to be parents' sense of competence measured by the Parental Sense of Competence Scale (PSOC)(37). The PSOC is a 17-item questionnaire showing good validity across several studies and widely used as a measure of parenting self-esteem(38, 39), collected at baseline and at 12-months post-randomisation.

The following potential *secondary intervention outcome measures* were incorporated into a user-friendly questionnaire booklet (around 250 items) and collected at baseline and at follow-up interviews:

- Eyberg Child Behaviour Inventory (ECBI)(40): measure of behaviour problems of children aged 2-16 to be completed by parents or carers;
- SDQ(41): self-report measure of behaviour of children aged 2-17 to be completed by parents/carers/teachers/children aged 11yrs or older;
- General Health Questionnaire (GHQ)(1): self-report measure of common psychiatric conditions to be completed by parents or carers;
- ADHD Symptom Rating Scale-version 1 (ASRSv1)(42): symptom checklist consisting of 18 DSM-IV criteria to be completed by practitioners with regards parents;
- Parenting Daily Hassles Scale (PDHS)(43): 18-item self-report measure of the frequency and intensity of 20 potential parenting 'hassles' to indicate parents' stress;
- Goal Based Outcomes (GBO) from the Child Outcomes Research Consortium (CORC)(44):
- EQ-5D-5L (45): a self-report measure of parent quality of life;
- PedsQL (46): a self-report measure of child quality of life;

- Participant & child health service use; a checklist of inpatient & outpatient services accessed within the last 12 months.

Analysis

A simple descriptive analysis of numbers of families recruited and retained relative to the eligible / approached population was conducted. Between-group change in each measure after adjustment for baseline was analysed using analysis of covariance (ANCOVA). This allowed exploration of preliminary effects of the intervention and will help decide on the sample size needed for a future definitive RCT. All analyses were overseen by the Trial Statistician.

Ethical review

The trial was reviewed by the North of Scotland Research Ethics Committee (18/NS/0124) and approved on 13 December 2018.

RESULTS

Recruitment and Retention

Thirty participants were recruited to this trial, fourteen were randomised to PInC and sixteen to IY. Recruitment and retention data are presented in Figure 1.

Fig. 1: CONSORT flow diagram

Sample Characteristics

Referring to Table 1, the socioeconomic spread of the sample was skewed towards those living in areas of higher deprivation and 41% of parents exhibited ADHD symptoms (i.e., endorsed $\geq$4 items on the opening screener questions) on the ASRS screening tool. There was little difference evident between groups at baseline.

Table 1. Parent characteristics

				STUDY ARM			
		All (30)		PInC (14)		IY (16)	
**Age (years)** Mean (SD)		39.1	(7.2)	38.8	(7.0)	39.3	(7.5)
**Gender** N (%)	Male	1	(3)	1	(7)	0	(0)
	Female	29	(97)	13	(93)	16	(100)
**Socioeconomic deprivation (SIMD*)** N (%)	Q1 (most deprived)	7	(23.3)	3	(21.4)	4	(25.0)
	Q2	12	(40.0)	7	(50.0)	5	(31.2)
	Q3	3	(10.0)	1	(7.1)	2	(12.5)
	Q4	4	(13.3)	3	(21.4)	1	(6.2)
	Q5 (least deprived)	4	(13.3)	0	(0.0)	4	(25.0)
**Parent ADHD symptoms (ASRS Part A)** N (%)	Yes	12	(41.4)	7	(50.0)	5	(33.3)
	No	18	(62.1)	7	(50.0)	10	(66.7)

* Scottish Index of Multiple Deprivation 2020(SIMD)(47)

Openness to randomisation

No potential participants cited randomisation as a reason for non-participation during recruitment phone-calls with the research team (see Fig. 1). Findings from qualitative interviews suggest that 18 of 24 participants interviewed were generally accepting of the principle of randomisation, and were motivated by a belief that attendance at any parenting programme would be of benefit to them. The fact that five participants wanted to attend the group to which they were not randomised (i.e. IY participants attending Parents InC groups) suggests that unwillingness to randomisation was present though not expressed (see Table 2). Interview data recorded the reasons for requested referral to non-randomised group as a) time constraints (a preference for Parents InC as taking less time), and b) ADHD focus of programme (Parents InC was preferred by some due to its focus on ADHD).

Table 2. Randomisation and group attendance summary

	N	Proportion
Participants consented & randomised	30	
Participants attended randomisation group (out of participants randomised)	17	56.7%
Participants requested non-randomisation group (out of participants randomised) (n=5 randomised to IY, n=0 randomised to Parents InC)	5	13.3%
Participants attended both groups (out of subjects randomised)	1	3.3%
Participants did not attend any group (out of subjects randomised)	7	23.3%

Practitioner referral

Referrer interview data indicated a degree of reluctance to refer based on concerns that referral to the research trial may impact therapeutic relations and perceived irrelevance to certain patients. Others cited workload (falling off their agenda, forgetting to refer) and uncertainty over professional remit as barriers to referral. Of the fifty-two families assessed for eligibility, only seven (13.5%) were referred directly by paediatricians/psychiatrists following assessment, and forty-five (86.5%) were already referred to Parents InC and were on a waiting list, and responded to invitation letters from the clinical team.

Feasibility & Acceptability of research procedures and measures

Table 3 demonstrates the mean number of contacts (telephone, text message and letter) per participant from the point of expression of interest to follow up data collection. The quantitative follow-up assessments took the highest number of contacts per participant (mean = 7.4) and, despite researcher concerns that this may be intrusive for participants, overall interview data suggested participants were happy with this level of contact.

> *"Yes, so the phone call and the text messages are fine, but I do appreciate that sometimes I am a bit hard to get a hold of because of work and stuff, but it has been fine, yeah… Aye, definitely; the reminder texts are great!" (female parent 1, follow-up interview).*

Table 3. Mean number of contacts per participant		
	No. of contacts	
	Total (n participants)	Mean (SD) per participant
**Quantitative baseline**	99 (30)	3.3 (1.9)
**Qualitative baseline**	139 (29)	4.8 (3.9)
**Quantitative Follow up**	221 (30)	7.4 (6.5)
**Qualitative Follow up**	90 (21)	4.3 (3.2)
**Total**	549	18.3 (9.7)

Participant motivation to engage with the research was explored during interviews, the key factor being the length of the child's journey to diagnosis; parents reported that having waited so long for a diagnosis, they were willing to participate in any activity which would support their child's ongoing development.

The acceptability of research procedures centred around three key themes during interview:

1) researcher approach: Participants commented on the importance of researchers' empathic interactions and flexibility of data collection methods (particularly relating to time of day/number of sessions and location of data collection). Both phone and face-to-face interviews were reported to be acceptable to qualitative interview participants.

> *"…it was fine, and you came to the house last time, that was absolutely fantastic as I didn't have to go anywhere, it worked round me, it was relaxed, same as this time, it has just been absolutely fine." (female parent 2, follow-up interview).*

2) study information: The majority of participants felt the study was well explained and facilitated by the research team.

"(…did it go to plan for you? Was it what you expected?) Yeah, it was and more. I think we had lots of support overall, any questions could get answered at any time and we've been given enough information…" (female parent 3, follow-up interview).

3) timing with intervention: Due to waiting times for randomised programmes 1 parent opted out of attending a programme, and 2 others attended programmes they weren't randomised to while waiting.

"I was quite happy to go on them and I was looking forward to it, but then it took too long as in it took too long to start with, to come round, so I think we must have waited may be almost ten months before we actually got put on a course and then obviously it was then for quite a long period of time, I think it was 14 weeks, and it was over lunch time, and it was for may be about two hours, and that didn't really suit us…" (female parent 4, follow-up interview).

Participants' views of the research measures were generally positive, and many found the questions familiar from clinical diagnostic interviews. Some participants reported feeling emotional or upset by discussing family medical history and detailing the everyday challenges they face in parenting their child with ADHD, though this did not cause sufficient distress to prevent their ongoing participation.

Parenting Sense of Competence Scale (PSOC) results at follow-up

Data from the Parenting Sense of Competence scale at baseline and follow-up for each intervention arm is reported in Table 4. Regression analysis suggests that participants who were randomised to the PInC group reported a higher sense of parenting competency following intervention than those randomised to the IY group.

Statistical power for a future definitive RCT

Recruitment and retention was reasonably good in this study, but 43% of participants did not go on to receive the intervention they were randomised to. Although we would seek to mitigate the

possibility of participants opting for the comparison intervention in a definitive trial (see limitations), we have chosen to retain a conservative approach to sample size calculation. We estimate that it would be feasible to detect 0.5 of a SD change in PSOC scores at 90% power with 191 participants in each arm. Based on our recruitment and follow-up rates, we would expect to have to recruit around 500 participants to reach this figure. A future trial could potentially stratify using variables such as parental ADHD status (ASRS cut-off) and SIMD.

Table 4. PSOC data from participants with baseline and follow-up data only

		Randomised intervention		
		All	PInC	IY
	N_{OBS}	18	9	9
**Baseline**	Mean (SD)	62.5 (7.0)	64.2 (8.0)	60.7 (5.8)
**Follow-up**	Mean (SD)	65.5 (7.1)	68.9 (7.1)	62.1 (5.7)
**Change from baseline**	Mean (SD)	3.0 (6.9)	4.7 (6.6)	1.4 (7.3)

Linear regression analysis predicting change from baseline from randomised intervention group adjusting for baseline PSOC, child age and gender

	Adjusted mean difference (95% CI)	p-value
**IY vs. PInC**	-7.44 (-14.14, -0.74)	0.049

All psychometric results at follow-up

A summary of all psychometric mean scores is presented in supplementary table S1.

Overall, the parent focussed outcomes (PSOC and parental GHQ) suggest the largest effects between intervention arms. Child-focussed outcome measures and quality of life suggested little difference between groups.

Economic summary

Costs per group were calculated at £14,957 for PInC; £26,489 for IY. In terms of associated health service costs: IY participants had more outpatient visits in 12 months post-randomisation, but use of other services was similar across arms.

DISCUSSION

The findings of this feasibility study support the intention of exploring the research questions in a future substantive randomised controlled trial (RCT). The trial has demonstrated reasonable recruitment and excellent retention rates. Participant feedback on research procedures and methods was positive, and qualitative interviews provided a rich source of learning points to take forward into a future definitive trial. The focus of this trial has been on parent outcomes following group intervention; this focus is upheld by results suggesting the largest between-group effect sizes on the parental sense of competence scale and parental General Health Questionnaire.

As both intervention groups were part of usual services within NHS Fife, recruitment to the research trial was dependent on referrals from NHS staff / access to the Parents InC waiting list. Interview data demonstrated a level of gate-keeping from referring practitioners based on misconceptions about the trial and heavy workloads resulting in de-prioritising of referrals. In a definitive RCT, the trial research team would retain full responsibility for recruitment by negotiating access to routine data to define the target population, as has been successful in other RCTs we have conducted (36). Whilst evaluating ongoing health services can be financially beneficial, there are doubtless compromises made to the integrity of the research project.

A positive aspect to recruitment was the interview method used in the informed consent and baseline measures process. Although this may be an intensive method, it may be a worthwhile investment in the context of this participant group and service setting.

It is interesting to note that the theme of gate-keeping within health care systems and systemic barriers to engagement in research and health services is often acknowledged (48). The use of the term “hard to reach” has long been misused and conveniently places responsibility upon those families to whom the term applies; leaving the systems and institutions who coined the term on a moral high ground, having “done their bit” and produced a beautifully crafted yet inaccessible programme. The developer of the Incredible Years programme shared the following insight:

“Such families have been described as unmotivated, resistant, unreliable, disengaged, chaotic...unlikely candidates for this kind of treatment — in short, unreachable. However, these families might well describe traditional clinic-based programs as ‘unreachable’. Clinical programs may be too far away from home, too expensive, insensitive, distant, inflexible in terms of scheduling or content, foreign in terms of language...blaming or critical of their lifestyle. Perhaps this population has been ‘unreachable’ not because of their own characteristics, but because of the characteristics of the interventions they have been offered.” (49)

While recruitment to this trial was lower than anticipated, retention was far higher. Once participants were successfully engaged, they expressed willingness to participate and motivation to engage with both the quantitative and qualitative components of the trial, therefore we anticipate higher recruitment rates if access to the entire target population was made possible through access to routine data. An interesting feature in participant motivation to engage was the length of the child’s journey to diagnosis; parents reported that having waited so long for a diagnosis, they were willing to participate in any activity which would support their child’s ongoing development.

Limitations

As discussed previously, the sample size for this feasibility study was small due to reliance on practitioner referral and staffing issues. Parents InC is the first-line parenting programme for parents of a child with ADHD in NHS Fife. This intervention was therefore available to all participants, even if

randomised to Incredible Years, allowing participants to request re-referral to Parents InC during the trial and this might have biased our findings regarding participant preferences. Not only did this result in a low sample size, only 57% (17/30) of those randomised received the intervention they were randomised to. This may be unlikely in a different trial context, but does impact on the application of these findings to the planning of a definitive trial. A future trial would be best placed within a setting where no or very limited current service provision exists, so that there is no ethical requirement to allow participants to select either intervention, regardless of their study allocation. Finally, the need to only include those with sufficient English language proficiency limits the generalisability of these findings: a future trial should consider how best to address this within the target population.

Conclusions

In a public healthcare system, it is imperative that treatments and interventions are both efficacious and cost-effective. The current research indicates that parental sense of competence and general health may be enhanced by attendance at a Parents InC group, and further research is required to explore this effect on a larger scale. Despite personal and situational barriers, populations such as reported here are not “hard” to reach and are often highly motivated to engage in services and research - it is our role as clinicians and researchers to commit to removing those barriers which inhibit their engagement.

ACKNOWLEDGEMENTS

We are extremely grateful to the parents who gave their time to take part in research assessments and interviews. Our Trial Steering Committee, including Geraldine Mynors, Dr Christine Puckering,

and Linda Frazer (Patient and Public Involvement representative) offered valuable discussion and reflection. Thanks to Fatene Ismail, Charlotte Paterson, and Paula Regener who previously worked on the trial. We appreciate the input of Martina Messow for writing the original Statistical analysis plan and supervising descriptive and outcome analyses, and of Ting Ting Wu for conducting health economic analysis. Our co-investigators helped shaped the design of the study: Nick Watson, Rachael Wood. Finally, thanks to Dr Hilary Maddox, Consultant Clinical Psychologist and Head of Child and Family Psychology Service, NHS Fife, and Gordon Brown, Clinical Lead and CEO of ADHD Direct, who developed the Parents InC programme and provided information on the theory of change. Dr Maddox also hosted the study within her service.

REFERENCES

1. Goldberg DP, Gater R, Sartorius N, Ustun TB, Piccinelli M, Gureje O, et al. The validity of two versions of the GHQ in the WHO study of mental illness in general health care. Psychological medicine. 1997;27(1):191-7.
2. Rasmussen P, Gillberg C. Natural outcome of ADHD with developmental coordination disorder at age 22 years: a controlled, longitudinal, community-based study. Journal of the American Academy of Child and Adolescent Psychiatry. 2000;39(11):1424-31.
3. Rutter M, Kim-Cohen J, Maughan B. Continuities and discontinuities in psychopathology between childhood and adult life. J Child Psychol Psychiatry. 2006;47(3-4):276-95.
4. Faraone SV, Asherson P, Banaschewski T, Biederman J, Buitelaar JK, Ramos-Quiroga JA, et al. Attention-deficit/hyperactivity disorder. Nature reviews Disease primers. 2015;1:15020.
5. Matza LS, Paramore C, Prasad M. A review of the economic burden of ADHD. Cost Effectiveness and Resource Allocation. 2005;3(1):5.
6. Gillberg C. The ESSENCE in child psychiatry: Early Symptomatic Syndromes Eliciting Neurodevelopmental Clinical Examinations. Research in Developmental Disabilities. 2010;31(6):1543-51.
7. King S, Griffin S, Hodges Z, Weatherly H, Asseburg C, Richardson G, et al. A systematic review and economic model of the effectiveness and cost-effectiveness of methylphenidate, dexamfetamine and atomoxetine for the treatment of attention deficit hyperactivity disorder in children and adolescents. Health technology assessment (Winchester, England). 2006;10(23):iii-iv, xiii-146.
8. Psychogiou L, Daley D, Thompson M, Sonuga-Barke E. Testing the interactive effect of parent and child ADHD on parenting in mothers and fathers: A further test of the similarity-fit hypothesis. British Journal of Developmental Psychology. 2007;25(3):419-33.
9. Modesto-Lowe V, Danforth JS, Brooks D. ADHD: does parenting style matter? Clin Pediatr (Phila). 2008;47(9):865-72.
10. Danforth JS, Anderson LP, Barkley RA, Stokes TF. Observations of parent-child interactions with hyperactive children: Research and clinical implications. Clinical psychology review. 1991;11(6):703-27.
11. DuPaul GJ, McGoey KE, Eckert TL, VanBrakle J. Preschool children with attention-deficit/hyperactivity disorder: impairments in behavioral, social, and school functioning. J Am Acad Child Adolesc Psychiatry. 2001;40(5):508-15.
12. Johnston C. Parent characteristics and parent-child interactions in families of nonproblem children and ADHD children with higher and lower levels of oppositional-defiant behavior. J Abnorm Child Psychol. 1996;24(1):85-104.
13. Keown LJ, Woodward LJ. Early parent-child relations and family functioning of preschool boys with pervasive hyperactivity. J Abnorm Child Psychol. 2002;30(6):541-53.
14. Bali P, Sonuga-Barke E, Mohr-Jensen C, Demontis D, Minnis H. Is there evidence of a causal link between childhood maltreatment and attention deficit/hyperactivity disorder? A systematic review of prospective longitudinal studies using the Bradford-Hill criteria. JCPP Advances.n/a(n/a):e12169.
15. Banks T, Ninowski JE, Mash EJ, Semple DL. Parenting Behavior and Cognitions in a Community Sample of Mothers with and without Symptoms of Attention-deficit/Hyperactivity Disorder. J Child Fam Stud. 2008;17(1):28-43.
16. Harvey E, Danforth JS, McKee TE, Ulaszek WR, Friedman JL. Parenting of children with attention-defecit/hyperactivity disorder (ADHD): the role of parental ADHD symptomatology. Journal of attention disorders. 2003;7(1):31-42.
17. Murray C, Johnston C. Parenting in mothers with and without attention-deficit/hyperactivity disorder. J Abnorm Psychol. 2006;115(1):52-61.

18. Weiss M, Hechtman L, Weiss G. ADHD in parents. J Am Acad Child Adolesc Psychiatry. 2000;39(8):1059-61.
19. Johnston C, Mash EJ, Miller N, Ninowski JE. Parenting in adults with attention-deficit/hyperactivity disorder (ADHD). Clinical psychology review. 2012;32(4):215-28.
20. Excellence NNIfHaC. Attention deficit hyperactivity disorder: diagnosis and management. 14 March 2018. Available from: https://www.nice.org.uk/guidance/ng87/chapter/recommendations#diagnosis.
21. Stattin H, Enebrink P, Ozdemir M, Giannotta F. A national evaluation of parenting programs in Sweden: The short-term effects using an RCT effectiveness design. Journal of consulting and clinical psychology. 2015;83(6):1069-84.
22. Coates J, Taylor JA, Sayal K. Parenting Interventions for ADHD: A Systematic Literature Review and Meta-Analysis. Journal of attention disorders. 2015;19(10):831-43.
23. Sonuga-Barke EJ, Daley D, Thompson M. Does maternal ADHD reduce the effectiveness of parent training for preschool children's ADHD? J Am Acad Child Adolesc Psychiatry. 2002;41(6):696-702.
24. Chronis-Tuscano A, O'Brien KA, Johnston C, Jones HA, Clarke TL, Raggi VL, et al. The relation between maternal ADHD symptoms & improvement in child behavior following brief behavioral parent training is mediated by change in negative parenting. J Abnorm Child Psychol. 2011;39(7):1047-57.
25. Durand G, Arbone IS, Wharton M. Reduced organizational skills in adults with ADHD are due to deficits in persistence, not in strategies. PeerJ. 2020;8:e9844.
26. Smith E, Koerting J, Latter S, Knowles MM, McCann DC, Thompson M, et al. Overcoming barriers to effective early parenting interventions for attention-deficit hyperactivity disorder (ADHD): parent and practitioner views. Child: care, health and development. 2015;41(1):93-102.
27. Kazdin AE. Child, parent and family dysfunction as predictors of outcome in cognitive-behavioral treatment of antisocial children. Behav Res Ther. 1995;33(3):271-81.
28. Reyno SM, McGrath PJ. Predictors of parent training efficacy for child externalizing behavior problems – a meta-analytic review. Journal of Child Psychology and Psychiatry. 2006;47(1):99-111.
29. Koerting J, Smith E, Knowles MM, Latter S, Elsey H, McCann DC, et al. Barriers to, and facilitators of, parenting programmes for childhood behaviour problems: a qualitative synthesis of studies of parents' and professionals' perceptions. Eur Child Adolesc Psychiatry. 2013;22(11):653-70.
30. Cortis NaK, Ilan and Patulny, Roger. Engaging Hard-to-Reach Families and Children, Occasional Paper No. 26. In: FaHCSIA, editor. (2009).
31. Stone P, Maddox H. Parents Inc®: Assessing the clinical effectiveness of a Group Parenting Programme for Parents of children with Attention-Deficit / Hyperactivity Disorder. 2015.
32. Jones K, Daley D, Hutchings J, Bywater T, Eames C. Efficacy of the Incredible Years Programme as an early intervention for children with conduct problems and ADHD: long-term follow-up. Child: care, health and development. 2008;34(3):380-90.
33. Barlow J, Smailagic N, Huband N, Roloff V, Bennett C. Group-based parent training programmes for improving parental psychosocial health. The Cochrane database of systematic reviews. 2014(5):Cd002020.
34. Gardner F, Leijten P, Mann J, Landau S, Harris V, Beecham J, et al. Public Health Research. Could scale-up of parenting programmes improve child disruptive behaviour and reduce social inequalities? Using individual participant data meta-analysis to establish for whom programmes are effective and cost-effective. Southampton (UK): NIHR Journals Library; 2017.
35. Foubister L, Rennie F, Williams J. Parents in Control: Parental perceptions of problem behaviors before and after attending an ADHD-specific parent-training program. Journal of child and adolescent psychiatric nursing : official publication of the Association of Child and Adolescent Psychiatric Nurses, Inc. 2020;33(1):30-7.
36. Minnis H. Evaluation of the New Orleans Intervention for Infant Mental Health (BEST?). 2011.

37. Gilmore L, Cuskelly M. Factor structure of the Parenting Sense of Competence scale using a normative sample. Child: care, health and development. 2009;35(1):48-55.
38. Garfield CF, Lee YS, Kim HN, Rutsohn J, Kahn JY, Mustanski B, et al. Supporting Parents of Premature Infants Transitioning from the NICU to Home: A Pilot Randomized Control Trial of a Smartphone Application. Internet interventions. 2016;4(Pt 2):131-7.
39. Trillingsgaard T, Maimburg RD, Simonsen M. The Family Startup Program: study protocol for a randomized controlled trial of a universal group-based parenting support program. BMC Public Health. 2015;15:409.
40. Eyberg SM, Ross AW. Assessment of child behavior problems: The validation of a new inventory. J Clin Child Psychol. 1978;7(2):113-6.
41. Goodman R. Psychometric properties of the strengths and difficulties questionnaire. Journal of the American Academy of Child and Adolescent Psychiatry. 2001;40(11):1337-45.
42. Kessler RC, Adler L, Ames M, Demler O, Faraone S, Hiripi E, et al. The World Health Organization Adult ADHD Self-Report Scale (ASRS): a short screening scale for use in the general population. Psychological medicine. 2005;35(2):245-56.
43. Crnic KA, Greenberg MT. Minor parenting stresses with young children. Child Dev. 1990;61(5):1628-37.
44. CORC. Goal Based Outcomes [Available from: http://www.corc.uk.net/outcome-experience-measures/goal-based-outcomes/.
45. Janssen MF, Pickard AS, Golicki D, Gudex C, Niewada M, Scalone L, et al. Measurement properties of the EQ-5D-5L compared to the EQ-5D-3L across eight patient groups: a multi-country study. Quality of life research : an international journal of quality of life aspects of treatment, care and rehabilitation. 2013;22(7):1717-27.
46. Varni JW, Seid M, Kurtin PS. PedsQL 4.0: reliability and validity of the Pediatric Quality of Life Inventory version 4.0 generic core scales in healthy and patient populations. Medical care. 2001;39(8):800-12.
47. Government S. Scottish Index of Multiple Deprivation 2020. In: Directorate HaSJ, editor. 2020.
48. Boag-Munroe G, Evangelou M. From hard to reach to how to reach: A systematic review of the literature on hard-to-reach families. Research Papers in Education. 2012;27(2):209-39.
49. Webster-Stratton C. Parent Training with Low-Income Families. In: Lutzker JR, editor. Handbook of Child Abuse Research and Treatment. Boston, MA: Springer US; 1998. p. 183-210.

Figure

Click here to access/download;Figure;SPARCLAR CONSORT flow diagram Dec23.docx

SPARCLAR CONSORT flow diagram Jan 2021

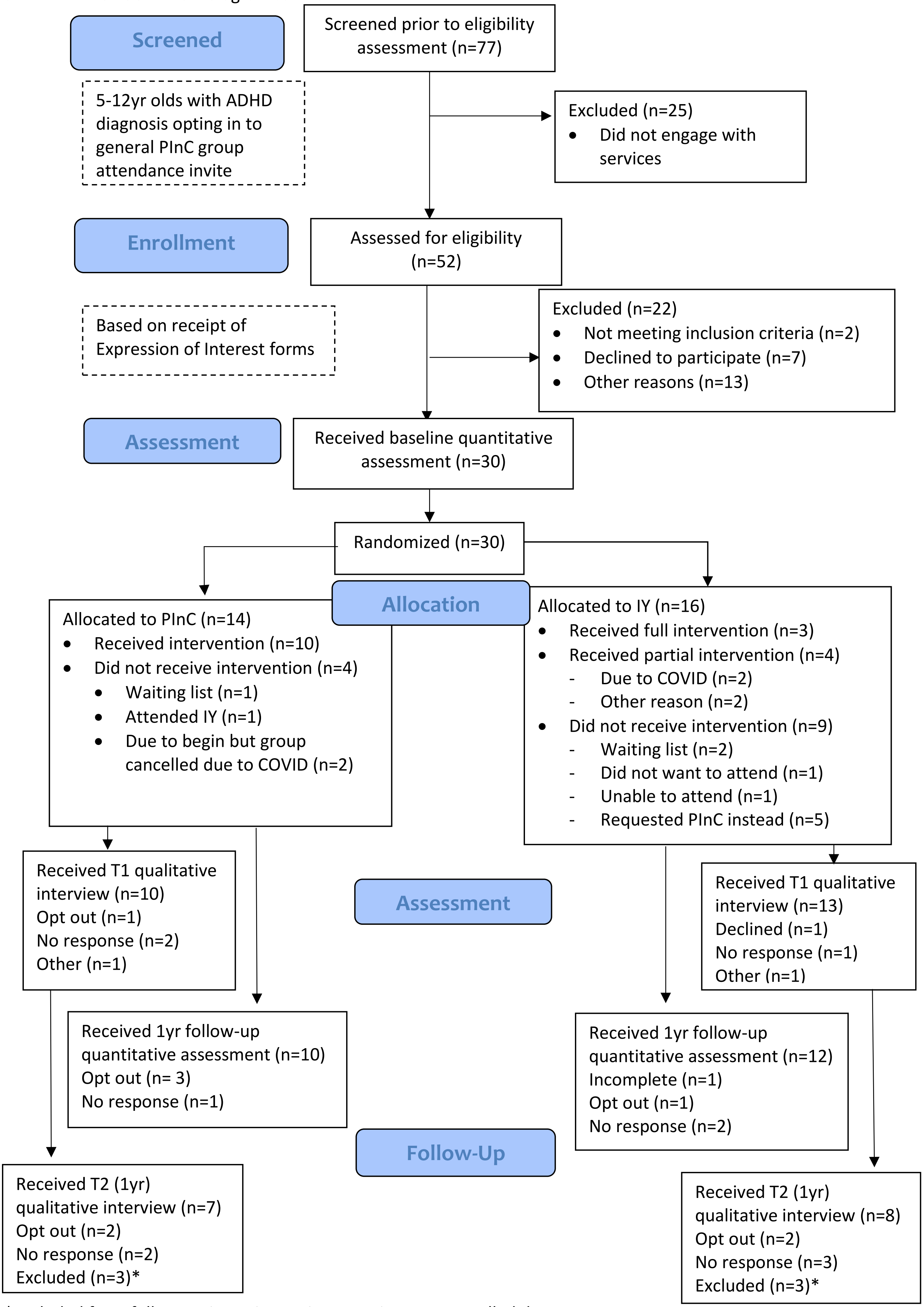

*excluded from follow-up interview as intervention was cancelled due to COVID19
