# Supplementary material for: Families with neurodevelopmental diagnoses are not ‘Hard to Reach’: Findings from a feasibility trial comparing parenting programmes for parents of children with ADHD
Source: PLoS One. 2025 Sep 17;20(9):e0323959. doi: 10.1371/journal.pone.0323959 (PMC12443243; doi:10.1371/journal.pone.0323959)
Supplement: Attachment — Submitted filename: Response to reviewers.pdf [file pone.0323959.s005.pdf]

University of Glasgow
School of Health & Wellbeing

16 January 2025

Dear Mu-Hong Chen

Re: PONE-D-24-36397 Families with neurodevelopmental diagnoses are not 'Hard to Reach': Findings from a feasibility trial comparing parenting programmes for parents of children with ADHD

We very much appreciate the constructive comments from reviewers on our manuscript. We have addressed each of their points in turn in the table below. We have also responded to the reviewer annotations on the provided pdf. In addition to the requested changes, we have implemented a few edits for typographical errors and removal of redundant text and have added two supplementary files. All documents have been uploaded in the appropriate format.

We have also changed our view on making the data available via open access. Whilst we subscribe to the principle of open access to research data, upon reflection we have concerns about the identifiability of this small sample. Anonymisation will not be sufficient to protect the confidentiality of these participants given the available details about time and location of the research. We trust this is acceptable to the editors.

Response to reviewers' comments

	Reviewer comment	Author response
R1.1	Clarification of Objectives:<br>o Issue: The manuscript lacks a clear and detailed explanation of the study objectives.<br>o Recommendation: Revise the introduction to explicitly state the research questions. Clearly articulate what the study aims to demonstrate regarding the reach and effectiveness of different parenting programs.	We have articulated our specific aims on lines 95-104. This study was a feasibility trial so was not seeking to demonstrate effectiveness of any parenting programme. The aim was rather to explore the feasibility of conducting such a large trial, thus the aims are stated in way that reflects the exploratory nature of the work. We provide further detail on our recruitment aim (b) in the Methods section (lines 123-128).
R1.2	Methodology Details:<br>o Issue: There is insufficient detail about the study design, participant selection, and intervention procedures.<br>o Recommendation: Provide a more comprehensive description of the study design, including randomization procedures, inclusion and exclusion criteria, and detailed descriptions of the parenting programs compared. This will help in assessing the robustness of the methodology.	Our inclusion criteria were as stated at line 114, with exclusion criteria given in the subsequent paragraph. No further detail exists.<br>Randomisation method is given on lines 148-150, including both practical method (IRVS at the clinical research facility) and randomisation approach (1:1 in permuted blocks of 4 with no stratification or minimisation).<br>The interventions were described in the Introduction, although this detail could be moved to methods. As this was not an effectiveness trial but a feasibility trial, it was not deemed necessary to include further detail.

	Reviewer comment	Author response
		We have reviewed the whole methods section and cannot see any further room for detail to be added. We welcome any specific feedback regarding the areas referred to above.
R1.3	3. Statistical Analysis:<br>o Issue: The statistical methods used are not adequately described, and there is a lack of justification for the chosen analyses.<br>o Recommendation: Elaborate on the statistical techniques employed, including how they were selected and why they are appropriate for the data. Provide justification for any assumptions made and discuss how missing data were handled.	The statistical methods were in line with the aims of the study, namely to assess the feasibility of carrying out a trial of these two interventions. Feasibility measures are reported descriptively, and ANCOVA for continuous outcome measures collected at baseline and follow is standard practice for randomised trials, thus we feel that no more elaboration is required. We have added a statement about the handling of missing data (line 202), and the use of a pre-specified Statistical Analysis Plan (which we have uploaded as a supplementary file).
R1.4	Results Presentation:<br>o Issue: The presentation of results is somewhat disorganized and lacks coherence.<br>o Recommendation: Reorganize the results section to clearly present the findings for each parenting program in a systematic manner. Use tables and figures to summarize key results and make comparisons more accessible.	We have added an introduction to the Results section to remind the reader about the aims of the study (lines 208-209). Otherwise, we are unclear as to how the Results could be better presented. We have provided clear headings, and have covered all areas as stated in the aims. We have used tables and figures inasmuch as the data warrant them. We have added a table (Table 2) of child characteristics to the manuscript, and a table of full psychometric data (Table S1. Between-group difference secondary outcome measures) in a supplementary file. We welcome any specific feedback regarding the areas referred to above.
R1.5	Discussion and Interpretation:<br>o Issue: The discussion does not sufficiently interpret the findings in the context of existing literature and does not address potential limitations.<br>o Recommendation: Expand the discussion to contextualize the findings within the broader literature on ADHD and parenting programs. Address the study's limitations in detail and suggest how these limitations might affect the interpretation of the results.	Given that the primary outcome of this study was on the feasibility measures, we have chosen to focus on this literature in the Discussion. The Limitations sub-section also clearly addresses the study limitations in this respect. We have expanded a little on the literature in Discussion (see 1.7 below) but with a focus on the feasibility of conducting a larger trial in this context (rather than the effectiveness of parenting programmes).
R1.6	Implications for Practice:<br>o Issue: The implications for practice and policy are not clearly articulated.<br>o Recommendation: Articulate the practical implications of the findings for clinicians and policymakers. Provide specific recommendations based on the results, and discuss how these recommendations might	Given that this was a feasibility trial, it would be premature to comment on implications for clinical practice based on the findings. The primary implication from these results is that larger studies can and should be done, so that the best interventions can be made available in the future.

	Reviewer comment	Author response
	influence future interventions for families with neurodevelopmental diagnoses.	
R1.7	References and Literature Review:<br>o Issue: The literature review is outdated and does not incorporate recent studies.<br>o Recommendation: Update the literature review to include recent research relevant to ADHD parenting programs. Ensure that all cited references are current and pertinent to the study.	Thank you for this feedback. We have welcomed the opportunity to update many of the citations we have used, as well as to edit a little for brevity / succinctness.
R1.8	Formatting and Presentation:<br>o Issue: The manuscript requires formatting adjustments to meet journal guidelines.<br>o Recommendation: Revise the manuscript to adhere to the journal's formatting requirements, including section headings, font size, and citation style.	We have reviewed the manuscript and made some small corrections. We had already used clear headings (2 levels), appropriate standard font size, and Vancouver citation style. Please do let us know if we have overlooked something specific.
R1.9	Conclusion:<br>The manuscript presents important findings that could contribute significantly to the field of parenting programs for families with children with ADHD. However, substantial revisions are required to enhance the clarity, methodological rigor, and overall impact of the study. Addressing the points outlined above will strengthen the manuscript and better position it for publication.<br>Recommendation:<br>Revise and resubmit for further review.	We have presented a rigorously-conducted feasibility trial, where the focus is on recruitment, retention, and acceptability. We have purposely not reported the full range of measures used as these were included primarily as a means of assessing acceptability. With a sample size as small as this, we cannot present the outcome of these measures as providing any indication of the comparative effectiveness of the two interventions. Rather, we have presented the analysis of the proposed primary outcome measure for a definitive trial as a point of interest to the reader. To over-report the other measures would be to over-state the findings and detract from the primary purpose of the study. We have now added a table of full psychometric findings as a supplementary file only (Table S1. Between-group difference secondary outcome measures).
R1.10	Additional Comments:<br>• Ensure that all ethical considerations are clearly addressed.<br>Consider adding a summary of the study's contributions to the field at the end of the discussion.	We have provided information about ethical review and have outlined our approach to inclusion very clearly. We believe we have provided all relevant information – please let us know of any specific information that is missing.<br><br>We have amended our Conclusion to make it more clearly focused on the primary outcome of the study.
R2.1	One key concern is the lack of detailed information about the children's baseline psychiatric conditions in each group, particularly the severity of ADHD and any comorbidities such as anxiety, depression, or autism spectrum disorder. Understanding	We do not have data on children's full psychiatric comorbidities. Parents were asked if their child has any other neurodevelopmental diagnosis, but there is no detail as to which diagnosis/es the participants' children had. We agree this

	Reviewer comment	Author response
	the severity of the children's symptoms and the presence of any other conditions is essential because these factors can significantly influence treatment outcomes. For instance, if one group has children with more severe ADHD or additional comorbid conditions, it could confound the results and make it difficult to draw accurate conclusions about the relative effectiveness of the two parenting programs. Furthermore, the different target audiences of the two programs also pose a challenge when interpreting the results. As noted in the manuscript, IY addresses not only ADHD, but also oppositional defiant disorder (ODD), conduct disorder (CD), and anxiety. Meanwhile, Parents InC is more focused on ADHD specifically. Given that these conditions can significantly affect parenting dynamics and the child-parent relationship, comorbidities could be an important factor in explaining the differences in outcomes between the two groups. The study would benefit from a deeper exploration of these potential confounders, especially as IY may have broader applications and address a wider range of behavioral and emotional challenges.	will be essential information to have as part of a definitive effectiveness trial.<br>Whilst other diagnoses have not been detailed, we do have data on children's psychiatric symptoms using the SDQ and ECBI (now included as a supplementary file (Table S1. Between-group difference secondary outcome measures)). We chose not to include this in our manuscript as it is not the focus of the paper. We used these measures as a means of assessing the acceptability of them to parent participants, rather than to report on the results.<br>In this case, our primary outcome was in relation to feasibility of recruitment and retention and we have focused our reporting on this.<br>We agree with the reviewer's comments on potential confounding give the different scope of the two programs. This would need to be considered within a future definitive effectiveness trial.
R2.2	Additionally, as the study only reports on parent-focused outcomes like parental sense of competence (PSOC) and the General Health Questionnaire (GHQ), the children's treatment — particularly medications — is another important factor that is notably missing from the study. Pharmacological treatment is the first-line ADHD management for children over the age of six, and since both ADHD medication and behavioral interventions can independently influence outcomes, it would have been crucial to know the treatment regimens for the children in both groups. Without this information, it's difficult to ascertain whether any observed effects are due to the parenting interventions alone or influenced by pharmacological treatments that were not accounted for.	We have data on whether children were on medication, and which type. However, as above, the focus of this paper is not on the comparative effectiveness of the two interventions, but on the feasibility of the trial in terms of recruitment, retention, and acceptability of measures. We are able to snow that collecting this information was acceptable to parents, and any future trial should of course consider these variables in analysis.

We hope this response will allow our paper to be published in the near future.
Yours sincerely

Dr Lucy Thompson (on behalf of the authors)

CeDAR
Centre for Developmental Adversity and Resilience
