# Supplementary material for: Families with neurodevelopmental diagnoses are not ‘Hard to Reach’: Findings from a feasibility trial comparing parenting programmes for parents of children with ADHD
Source: PLoS One. 2025 Sep 17;20(9):e0323959. doi: 10.1371/journal.pone.0323959 (PMC12443243; doi:10.1371/journal.pone.0323959)
Supplement: Attachment — Submitted filename: Response to reviewers.docx [file pone.0323959.s006.docx]

5 March 2025

Dear Mu-Hong Chen

**Re: PONE-D-24-36397 Families with neurodevelopmental diagnoses are not ‘Hard to Reach’: Findings from a feasibility trial comparing parenting programmes for parents of children with ADHD**

We very much appreciate the further constructive comments from reviewers on our manuscript. We have addressed each of their points in turn in the table below.

**Response to reviewers’ comments**

|  | Reviewer comment | Author response |
| --- | --- | --- |
| R1.1 | The introduction could benefit from a clearer articulation of the specific research gap addressed by the study. While the importance of ADHD parenting programs is emphasized, the distinct advantages of comparing Parents InC and IY are less explicit. | We have added wording in lines 77-94 to make the research gap and rationale for comparing these specific interventions more clear. |
| R1.2 | Consider discussing broader implications for other neurodevelopmental conditions given the overlap in symptomatology such as Autism Spectrum Disorder. | Whilst there is considerable overlap in ASD and ADHD symptomology, for families where ADHD is clearly the most prominent and problematic condition it is important to have a service which has that specific focus. Ideally neurodevelopmental services will be established that include both a diagnosis-specific and more holistic approach to service provision, but the reality in the UK at present is that these tend to be set up in a more compartmentalised way, and interventions reflect this.  We haven’t added any comment to the paper at this stage as the main focus is on the feasibility aspect of the methodology, rather than broader implications regarding the interventions. This would of course need to be addressed within the development of a definitive RCT. |
| R1.3 | The rationale for excluding parents with low English proficiency could be elaborated further. | The funding limit for this study did not allow the inclusion of participants who would require a language translator. Similarly, the measures used may not necessarily be valid when used via an interpreter or with participants from other cultural backgrounds. This study is merely the first step in establishing proof of concept re. feasibility – a definitive trial would need to be better funded, more inclusive, and more able to ensure broader inclusion in a way that preserves research integrity. We have added wording to lines 407-408 to acknowledge this overtly. |
| R1.4 | While the randomization method is described, it would be helpful to clarify whether participants were blinded to their allocation and how this might have influenced outcomes. | Due to publicly-accessible information regarding usual care in the region where the research was conducted, as well as the difference in time commitment and nature of the two programmes, it was impossible for participants to be blinded to randomisation status. It is extremely rare for participants to be blinded in trials of social interventions of this nature, and to not fully inform participants of the nature of the interventions would be considered unethical. |
| R1.5 | The choice of the Parenting Sense of Competence Scale (PSOC) as the primary measure is appropriate but could be supplemented with observational measures of parent-child interaction for future trials. | Direct observation of parent-child interaction would add a dimension to the measures, but would only ever be secondary to the main outcomes of the trial. PInC aims to improve parent confidence and sense of competence specifically, and it may be difficult to engineer an appropriate observation paradigm with school-aged children that would provide useful data. |
| R1.6 | The sample size of 30 participants is acknowledged as a limitation. While this is typical for a feasibility study, discussing strategies to improve recruitment such as more robust outreach efforts or incentives) would strengthen this section. | We believe the majority of the Discussion section addresses this point. |
| R1.7 | Table 3 highlights participants requesting non-randomized groups. This preference deserves more exploration in the discussion, as it reflects on the acceptability of the interventions. | In lines 248-250 of the Results we give the specific reasons for participants requesting non-randomised groups, including the nature of the intervention. We also mention this presents a design limitation in lines 389-390. We do not believe further discussion is needed at this stage. |
| R1.8 | The discussion of barriers to recruitment and retention is insightful but could benefit from greater emphasis on potential systemic solutions such as improving practitioner referral pathways or addressing gatekeeping. | These issues are touched upon in the Discussion. We do not believe there is scope, based on the current small study, to expound in greater detail. |
| R1.9 | The authors critique the term "hard to reach" and advocate for a systems-level perspective. This is a compelling argument, but more concrete recommendations for service redesign would add practical value. | We have touched upon this in the Discussion. As above, however, we do not feel that making robust suggestions regarding service redesign is appropriate following a small-scale feasibility trial such as this. |
| R1.10 | The differences in session length and focus between Parents InC (6 sessions) and IY (14 sessions) should be more critically evaluated. Are these differences in program structure likely to impact scalability and accessibility? | The content added to the Introduction regarding the rationale for the use of these interventions (R1.1) goes some way to addressing this point. There is no doubt that the difference in logistical aspects of these interventions could have an impact on scalability and accessibility. We have added some content to the Discussion (lines 403-405) to elaborate on this. |
| R1.11 | While the authors acknowledge the limited generalizability due to language restrictions and reliance on existing services, further discussion on how these limitations can be mitigated in future research would be valuable. | We have made suggestions on lines 348-350 and 394-396 regarding mitigation of the reliance on existing services. We have added some wording to lines 407-408 regarding making a trial more accessible to those without English language proficiency. |
| R1.12 | The CONSORT flow diagram is helpful but could be improved by visually emphasizing the drop-out points and reasons. | The CONSORT flow diagram has been developed according to the 2016 guidance (<https://www.bmj.com/content/355/bmj.i5239>). It is not clear what such an emphasis would add. |
| R1.13 | Tables summarizing qualitative themes could be added to complement the narrative findings. | We do not intend to present the full qualitative findings within this paper. A further paper will provide a more detailed presentation and analysis of the interview data. |
| R1.14 | Ensure consistent use of terminology such as "Parents InC" vs. "PInC". | We have restricted use of the PInC acronym to tables. Otherwise Parents InC is used, as the most common term used when mentioning the intervention in practice. |
| R1.15 | Some references are slightly dated such as DSM-5 reference from 2022. Consider incorporating more recent studies to highlight ongoing developments in ADHD research and interventions. | The reference to the DSM is the most up-to-date version, which guides clinical practice. As this trial was completed in 2021, it would not make sense to add anything more up-to-date in terms of the rationale for this research.  In the Discussion, we have centred the content on the feasibility aspect, i.e., the main focus of the study. Here we have included up-to-date literature (e.g., Bazier et al (2024)). We have added some comment on the nature of research on parenting support for families impacted by ADHD by means of providing context for future research (lines 375-379). |
| R2.1 | The rationale for comparing Parents InC to Incredible Years is well articulated; however, further explanation is needed regarding why these particular interventions were chosen. Was Parents InC already widely used? Why was it not compared to another ADHD-specific program? | See response to R1.1 above. |
| R2.2 | The recruitment and retention data are well presented; however, the study does not sufficiently explore the reasons behind the high dropout rate or participation in non-randomized groups. | This study did not experience a high dropout rate. Once recruited, retention was very good. We do not have full data on what prevented eligible participants from proceeding to study participation. The participation in non-randomised groups has been addressed in R1.7 above. |
| R2.3 | It would be helpful to also address how to use data obtained from this study to guide the future ECT to explore factors relevant to "hard to reach" and "gate-keeping" phenomena. | This will require specific consideration of the service context in which a definitive RCT is planned, as suggested in lines 369-371 – context-specific pre-protocol work will be required. |

We hope this response will allow our paper to be published in the near future.

Yours sincerely

Dr Lucy Thompson (on behalf of the authors)
